# Whole Genome Sequences of Three *Treponema pallidum* ssp. *pertenue* Strains: Yaws and Syphilis Treponemes Differ in Less than 0.2% of the Genome Sequence

**DOI:** 10.1371/journal.pntd.0001471

**Published:** 2012-01-24

**Authors:** Darina Čejková, Marie Zobaníková, Lei Chen, Petra Pospíšilová, Michal Strouhal, Xiang Qin, Lenka Mikalová, Steven J. Norris, Donna M. Muzny, Richard A. Gibbs, Lucinda L. Fulton, Erica Sodergren, George M. Weinstock, David Šmajs

**Affiliations:** 1 Department of Biology, Faculty of Medicine, Masaryk University, Brno, Czech Republic; 2 Human Genome Sequencing Center, Baylor College of Medicine, Houston, Texas, United States of America; 3 The Genome Institute, Department of Genetics, Washington University School of Medicine, St. Louis, Missouri, United States of America; 4 Department of Pathology and Laboratory Medicine, University of Texas Medical School at Houston, Houston, Texas, United States of America; University of Washington, United States of America

## Abstract

**Background:**

The yaws treponemes, *Treponema pallidum* ssp. *pertenue* (TPE) strains, are closely related to syphilis causing strains of *Treponema pallidum* ssp. *pallidum* (TPA). Both yaws and syphilis are distinguished on the basis of epidemiological characteristics, clinical symptoms, and several genetic signatures of the corresponding causative agents.

**Methodology/Principal Findings:**

To precisely define genetic differences between TPA and TPE, high-quality whole genome sequences of three TPE strains (Samoa D, CDC-2, Gauthier) were determined using next-generation sequencing techniques. TPE genome sequences were compared to four genomes of TPA strains (Nichols, DAL-1, SS14, Chicago). The genome structure was identical in all three TPE strains with similar length ranging between 1,139,330 bp and 1,139,744 bp. No major genome rearrangements were found when compared to the four TPA genomes. The whole genome nucleotide divergence (*d_A_*) between TPA and TPE subspecies was 4.7 and 4.8 times higher than the observed nucleotide diversity (π) among TPA and TPE strains, respectively, corresponding to 99.8% identity between TPA and TPE genomes. A set of 97 (9.9%) TPE genes encoded proteins containing two or more amino acid replacements or other major sequence changes. The TPE divergent genes were mostly from the group encoding potential virulence factors and genes encoding proteins with unknown function.

**Conclusions/Significance:**

Hypothetical genes, with genetic differences, consistently found between TPE and TPA strains are candidates for syphilitic treponemes virulence factors. Seventeen TPE genes were predicted under positive selection, and eleven of them coded either for predicted exported proteins or membrane proteins suggesting their possible association with the cell surface. Sequence changes between TPE and TPA strains and changes specific to individual strains represent suitable targets for subspecies- and strain-specific molecular diagnostics.

## Author Summary

Spirochete *Treponema pallidum* ssp. *pertenue* (TPE) is the causative agent of yaws while strains of *Treponema pallidum* ssp. *pallidum* (TPA) cause syphilis. Both yaws and syphilis are distinguished on the basis of epidemiological characteristics and clinical symptoms. Neither treponeme can reproduce outside the host organism, which precludes the use of standard molecular biology techniques used to study cultivable pathogens. In this study, we determined high quality whole genome sequences of TPE strains and compared them to known genetic information for *T. pallidum* ssp. *pallidum* strains. The genome structure was identical in all three TPE strains and also between TPA and TPE strains. The TPE genome length ranged between 1,139,330 bp and 1,139,744 bp. The overall sequence identity between TPA and TPE genomes was 99.8%, indicating that the two pathogens are extremely closely related. A set of 34 TPE genes (3.5%) encoded proteins containing six or more amino acid replacements or other major sequence changes. These genes more often belonged to the group of genes with predicted virulence and unknown functions suggesting their involvement in infection differences between yaws and syphilis.

## Introduction

Yaws is a treponemal disease found in rural communities of tropical regions of Africa, Asia, Oceania and South America, that predominantly affects children under 15 years of age. The causative agent of yaws, *Treponema pallidum* ssp. *pertenue* (TPE), was discovered at the same time [1] as the causative agent of syphilis, *Treponema pallidum* ssp. *pallidum* (TPA, [2]). The estimated prevalence of yaws is about 2 million cases worldwide [3], however, up to 50 million cases were estimated before 1952 when WHO established ‘The Global Yaws Control Program’. Between 1952–1964, more than 300 million people were treated with penicillin and the global prevalence of yaws declined significantly [4], [5]. Yaws was almost eliminated, however, local epidemics appeared in several areas in the 1970s [6]. Now, yaws is re-emerging in rural populations of Africa, Asia and South America. Since humans are the only known reservoir of yaws and because of the availability of cheap, single-dose penicillin treatment, eradication of this disease remains promising. In fact, renewed plans to eradicate yaws emerged recently [7].

TPE strains are closely related to syphilis causing TPA strains and the two subspecies cannot be distinguished by morphology, protein electrophoresis, bacterial physiology or host immune response [8], [9]. The genetic relationship between *pallidum* and *pertenue* subspecies was determined in 1980 by measuring the degree of their DNA sequence homology. The data indicated that TPE strain Gauthier and TPA strain Nichols were identical within the limits of resolution of this technique (about 2% of the genome differences; [10]). This led to reclassification of both organisms into a single species [11]. Several genetic differences between both subspecies have been already published including differences in the TP1038 gene at position 122 [12], [13], in the 16S rRNA gene (unpublished data, cited in [14]), in the 5′- and 3′-flanking regions of TP0171 [15], in the *gpd* gene (TP0257) at position 579 [16], in tp92 (TP0326) [17], and in *tprI* and *tprC* [18]. Newly performed analyses on strains belonging to *pallidum* and *pertenue* subspecies suggested that the genetic differences in the homologous chromosomal regions were less than 0.5% [19]. Such subtle differences need to be further characterized using high quality whole genome sequencing.

Yaws and syphilis are distinguished on the basis of epidemiological characteristics and clinical symptoms. Although both yaws and syphilis are multi-stage diseases, yaws, unlike syphilis, is mostly characterized by skin nodules, skin ulcerations, joint and soft tissue destruction and bone affections. However, in certain cases, yaws treponemes can also infect the central nervous system, cardiovascular system and fetus [20]. Yaws is an endemic disease of humid and warm rural areas that is transmitted through direct skin contact from an infected patient to a recipient (preferably among children) via a preexisting defect in the recipient's skin barrier. Syphilis is a sexually transmitted (and congenital) disease affecting adults and newborns worldwide. However, the transmission route of syphilis and yaws may reflect epidemiological differences rather than inherent differences between TPA and TPE strains [21]. Differences in the clinical manifestation and epidemiology of both diseases are likely to reflect primary genetic differences in the corresponding genomes.

In this communication, we compare three complete genome sequences of *T. p.* ssp. *pertenue* (Samoa D, CDC-2, Gauthier) to genomes of four *T. p.* ssp. *pallidum* strains (Nichols, DAL-1, SS14, Chicago). Genetic differences, which are consistently found between all studied strains of TPE and all studied strains of TPA, can differentiate the two pathogens at the molecular level and are candidates for important virulence factors of syphilitic treponemes. Additionally, the identified sequence changes represent suitable targets for specific molecular diagnostics.

## Materials and Methods

### Isolation of *Treponema pallidum* chromosomal DNA

*T. pallidum* ssp. *pallidum* (TPA) strain Nichols and *T. pallidum* ssp. *pertenue* (TPE) strains CDC-2, Gauthier and Samoa D were grown in rabbit testes, extracted and purified from testicular tissue using Hypaque gradient centrifugation [22], [23]. Chromosomal DNA was prepared as described previously [23].

### DNA sequencing and assembly of the TPE Samoa D genome

Whole genome DNA sequencing used a combined approach including Comparative Genome Sequencing (CGS, [24]), 454 pyrosequencing [25] and the Solexa approach [26]. Isolated genomic DNA from Samoa D strain was amplified using REPLI-g kit (QIAGEN, Valencia, CA, USA) and the amplified genomic DNA was used for CGS. CGS resulted in 904 identified single nucleotide changes (SNPs) when compared to the TPA Nichols reference genomic DNA. In addition, 102 genomic regions were recommended for dideoxy-terminator sequencing (DDT) due to increased sequence heterogeneity between tested and reference genomes. Parallel to CGS, unamplified Samoa D DNA was sequenced using the 454 technique on a GS20 apparatus (454 Life Sciences Corporation, Branford, CT, USA) to average depth coverage 27×. The 454 method had an average read length of 100 bp. These reads were assembled, with a Newbler assembler, into 154 contigs covering 98.6% of the reference TPA Nichols genome (AE000520.1). The Solexa sequencing technology (Illumina, San Diego, CA, USA) was used to improve accuracy of the complete genomic sequence resulting in an additional 75× coverage with an average read length of 36 bp. These reads were assembled, with a Velvet assembler [27], into 230 contigs. All discrepancies in CGS, 454 and Solexa sequences (i.e. 575 sites covered with 361 individual PCR products) were resequenced using the DDT method.

### DNA sequencing and assembly of the TPE CDC-2 genome

A combination of 454 sequencing (36× coverage, 101 bp average read length, 98.6% coverage of the AE000520.1 genome), Solexa sequencing (74× coverage, 36 bp average read length), and DDT technology was used for determination of the complete CDC-2 genome sequence using amplified genomic DNA. All nucleotide discrepancies between Samoa D and CDC-2 genome were confirmed using the DDT method.

### DNA sequencing and assembly of the TPE Gauthier genome

Amplified genomic DNA of TPE Gauthier was sequenced using 454 (26× coverage, 101 bp average read length, 1508 contigs, 92.6% coverage of AE000520.1 genome), and Solexa methods (65× coverage, 36 bp average read length, 902 contigs). However, due to substantial contamination of the TPE genomic DNA with rabbit DNA (data not shown) and more than 1500 gaps in the assembled genome sequence, chromosomal Gauthier DNA was amplified in 134 overlapping PCR intervals (*Treponema pallidum* intervals - TPI, described in [19], [28]) covering the entire treponemal genome using a GeneAmp® XL PCR kit (Applied Biosystems, Forster City, CA, USA). To avoid misassembly of sequentially related genes, equimolar PCR products were combined into four pools that were sequenced as different samples on a 454 platform and one sample on a Solexa platform. 454 and Solexa sequencing resulted in 50× coverage (average read length 233 bp, 78 contigs, 98.5% coverage of AE000520.1 genome) and in 206× coverage (35 bp average read length, 80 contigs assembled using Velvet; [27]), respectively. All gaps in the complete sequence were filled using the DDT method.

### Sequencing of *tpr* genes and regions with repetitive sequences

To avoid misassembly of sequentially related genes, several chromosomal loci were XL PCR amplified in all 3 TPE genomes and DDT sequenced. These loci included rDNA loci and several TPI regions (names of genes are shown in parentheses) including TPI11 (*tprC*), TPI12 (*tprD*), TPI25A (*tprE*), TPI25B (*tprF, tprG*), TPI32B (*arp*), TPI34 (TP0470), TPI48 (*tprI, tprJ*), TPI67B (*tprK*), and TPI78 (*tprL*) [19]. Small insert libraries from XL-PCR products were prepared and sequenced as described previously [29], [30]. Alternatively, XL PCR products were DDT sequenced using the primer walking method.

### Identification of genetic heterogeneity within TPE genomes

The Solexa reads belonging to individual genomes were used for identification of genetic heterogeneity within TPE genomes. In brief, individual Solexa reads were aligned to the final genome sequence using the Burrows-Wheeler Aligner (BWA) [31]. For downstream analyses and variant callings, the Samtools package was applied [32].

### Whole genome fingerprinting (WGF)

To verify final genome assemblies, whole genome fingerprints [19], [28] were compared to the *in silico* restriction enzyme analysis of each sequenced genome. Three enzymes, *Bam*H I, *Eco*R I and *Hin*d III, were used for restriction analysis of all amplicons. The use of other enzymes was optional depending on the length of restriction fragments and the availability of restriction target sites. The average error rate of WGF for *Treponema paraluiscuniculi* strain Cuniculi A genome was calculated previously [33] and corresponded to 27.9 bp (1.6% of the average fragment length) with a variation range between 0 and 132 bp.

### Gene identification and annotation

FgenesB (Softberry Inc., New York, USA), GeneMark [34] and Glimmer [35] were used independently for prediction of open reading frames (ORFs) in the Samoa D genome sequence. Visualization of gene prediction was performed using a Genboree system (www.genboree.org) and the CONAN database [36], [37]. To predict genes coding for tRNA, rRNA and non-coding RNAs, tRNAscan [38], RNAmmer [39] and Rfam [40] were used. DNA comparisons were performed using BLASTN and BLASTX algorithms. Protein sequences were analyzed using BLASTP versus the nr database at NCBI [41]. When appropriate, other predictive tools were used as described previously [42]. For proteins with unpredicted functions, a gene size limit of 150 bp was applied. For the CDC-2 and Gauthier gene annotations, predicted gene coordinates from Samoa D genome were adapted and recalculated. When needed, genes were newly predicted in some regions. In most cases, the original locus tag values of annotated Nichols genes were preserved in TPE orthologs. Genes newly predicted in TPE genomes were named according to their preceding gene with a letter suffix (e.g. a).

### Comparisons of whole genome sequences

Three TPE complete genome sequences were compared to the genomes of four TPA strains (Nichols, SS14, Chicago and DAL-1). The Nichols strain genome (AE000520.1) was completely sequenced by Sanger sequencing method (DDT) and coding regions (ORFs) were identified with compositional analysis [23], [43]. Comparative Genome Sequencing (CGS) approach and DDT method were used for sequencing of the SS14 strain (CP000805.1). Identification of ORFs and gene annotations were based on Nichols predicted gene coordinates that were adapted and recalculated [29]. Nichols and SS14 genome sequences were improved by 454 and Illumina resequencing (unpublished data). The Chicago genome (CP001752.1) sequencing was based on Solexa method, annotation was made by J. Craig Venter Institute Annotation Service [44]. A combination of 454 sequencing, Solexa method and Sanger sequencing was used for determination of the complete DAL-1 genome sequence (CP003115, unpublished results). Identification of ORFs and annotations were based on the predicted gene coordinates from the Nichols and Samoa D genomes.

Two approaches were used to determine genome differences between and within subspecies including a script based on Cross match software (unpublished) and whole genome alignments. Whole genome alignments were calculated using SeqMan, a Lasergene program package software (DNASTAR, Madison, WI, USA), with manual corrections. These methods were used for gene annotations and for calculation of nucleotide changes present in intergenic and coding regions. Both *tprD* and *tprK* genes were excluded from these calculations because of their high sequence diversity between TPA and TPE strains. Subsequently, nucleotide changes between studied subspecies and within them were analyzed using *DnaSP* software, version 5.10 [45]. Genetic relatedness was characterized by calculation of nucleotide diversity (π) within TPE and TPA strains. Alignment of TPE and TPA strains was used to calculate whole genome nucleotide divergence (*d_A_*). Complete TPE and TPA nucleotide sequences (including *tprD* and *tprK* genes) were used for these calculations.

### Gene classification

TPE genes were classified into eight groups according to their probable function and into four categories (Table 1) according to the number of amino acid changes in the corresponding proteins coded compared to TPA proteins. The categories included (1) genes encoding identical proteins, (2) proteins with single amino acid substitution, (3) proteins with two to five amino acid changes, and (4) proteins with more than 6 amino acid changes or multiple sequence changes (MSC). MSCs were defined as 15 or more dispersed amino acid substitutions and/or indels longer than 10 amino acids and/or truncated (results of frameshift mutations, nonsense and start codon changing mutations) and/or full length proteins (results of reverted frameshift mutations). The distribution of genes into categories and gene functional groups was statistically analyzed.

**Table 1:** Distribution of identified differences between TPA and TPE proteins encoded by different functional gene groups.

Functional gene group	No. of genes encoding identical proteins (%) statistical significance^a^	No. of genes encoding proteins with 1 aa substitution (%) statistical significance	No. of genes encoding proteins with 2–5 aa substitutions (%) statistical significance	No. of genes encoding proteins with 6 and more changes and/or MSC^b^ (%) statistical significance	Total no. of genes (%)
General metabolism	**125 (77.6)** -c	**26 (16.2)** -	**9 (5.6)** -	**1 (0.6)** -	**161 (100)**
Cell processes; Cell structure	**86 (68.8)** -	**33 (26.4)** p = 0.033	**5 (4.0)** -	**1 (0.8)** -	**125 (100)**
DNA replication, repair, recombination	**35 (68.6)** -	**11 (21.6)** -	**4 (7.8)** -	**1 (2.0)** -	**51 (100)**
Regulation; Transcription; Translation	**137 (79.2)** -	**32 (18.5)** -	**4 (2.3)** -	**0 (0.0)** -	**173 (100)**
Transport	**80 (70.8)** -	**22 (19.5)** -	**11 (9.7)** -	**0 (0.0)** -	**113 (100)**
Virulence; Potential virulence factor	**12 (37.5)** p<0.001	**6 (18.7)** -	**3 (9.4)** -	**11 (34.4)** p<0.001	**32 (100)**
Unknown	**217 (66.2)** p = 0.009	**64 (19.5)** -	**27 (8.2)** -	**20 (6.1)** p = 0.005	**328 (100)**
No of genes in the category	**692**	**194**	**63**	**34**	**983**

Genetic differences were characterized in 983 similarly annotated genes and pseudogenes in both *T. p.* ssp. *pertenue* (TPE; Samoa D, CDC-2, and Gauthier) and *T. p.* ssp. *pallidum* strains (TPA; Nichols, DAL-1, SS14, Chicago). Only changes present in all investigated TPE strains when compared to all investigated TPA strains are shown in this table.

a statistically significant results when compared to the genes encoding components of general metabolism. The p-value was corrected using Bonferroni correction for multiple comparisons to p = 0.008 and p-values lower than 0.008 were considered as statistically significant. p-values lower than 0.05 are also shown.

b MSC (major sequence changes) were defined as 15 or more dispersed amino acid substitutions, indels longer than 10 amino acids, truncated and full length proteins (results of frameshift and reverted frameshift mutations, nonsense and start codon changing mutations).

c not statistically significant.

### Statistical analyses

*STATISTICA* version 8.0 (StatSoft, Tulsa, OK, USA) was used for statistical calculations. In addition, an interactive calculation tool for chi-square tests of “goodness of fit” and independence was used [46]. The numbers of genes in four categories (classified according to number of amino acid changes in the corresponding proteins) of each functional gene group (Table 1) were compared to the gene numbers found in the general metabolism functional group. The p-value was corrected using the Bonferroni correction for multiple comparisons to p = 0.008 and p-values less than 0.008 were considered to be statistically significant.

### Type of selection

For genes encoding proteins with two or more amino acid changes, the codon-based test for estimation of selection type was calculated using the Kumar model [47] and *MEGA4* software [48]. Before prediction of selection type, the presence of possible recombination events was tested in whole genome alignments of TPE and TPA strains using the *Recombination Detection Program* version 3.44 (RDP3) [49]. Three methods implemented in the RDP3 program were used including the RDP method, the GENECONV [50] and the Maximum Chi-squared (MaxChi) method [51], [52]. Non-default settings comprised a maximum p-value of 0.01 and a Bonferroni correction. For the RDP method, a window size of 10 nt was used. For the MaxChi method, the number of variable sites per window was set to 30. Recombinant regions predicted by all three methods were considered as significant and the affected genes were omitted from further analyses. Gene sequences containing frameshift mutations were also removed from alignment and analyses. Genes with predicted positive or purifying selection were subsequently tested for possible intragenomic recombination events using BLAST search. Expected threshold was set to 50, word size to 16 and 20, respectively. TPE genes containing sequences from other part of the genome causing differences between TPA and TPE sequences were considered as genes containing recombinant sequences and were removed.

### Nucleotide sequence accession numbers

The genomes of TPE Samoa D, CDC-2, and Gauthier were deposited in the GenBank under the accession numbers CP002374, CP002375, CP002376, respectively.

## Results

### Whole genome sequencing, intrastrain variability in *Treponema pallidum* ssp. *pertenue* genomes, genomic parameters and genome annotation

The genome of *Treponema pallidum* ssp. *pertenue* (TPE) Samoa D was sequenced using 3 independent whole genome sequencing methods including comparative genome sequencing approach (CGS), pyrosequencing and Solexa sequencing (see Material and Methods). In addition, the Sanger sequencing method was used for finishing the complete genome sequence. The genomes of *T. pallidum* ssp. *pertenue* CDC-2 and Gauthier were sequenced using 2 methods, pyrosequencing and Solexa technology. CGS method was already used previously for sequencing of the SS14 genome [29] and the sequence of the strain Chicago was obtained by Solexa sequencing [44].

Analysis of the Solexa data revealed 8, 15, and 2 genome positions showing intrastrain variability in the TPE CDC-2, Samoa D, and Gauthier genomes, respectively. The most variable gene included TPE_0897 (5 positions in the CDC-2, 13 in the Samoa D, and 2 in the Gauthier genome). In addition, the CDC-2 genome showed heterogeneity within the TPECDC2_0313 (2 positions) and TPECDC2_0622 (1 position) genes, and the Samoa D genome in the TPESAMD_0110 (1) and TPESAMD_0134 (1) genes.

TPE Samoa D genome was annotated *de novo* and this annotation served as a reference for annotation of CDC-2 and Gauthier genomes. The following names TPESAMD, TPEGAU and TPECDC2 were assigned as gene names for the loci of Samoa D, Gauthier and CDC-2 strain, respectively. Gene names for the loci of all TPE strains start with the prefix TPE. The summarized genomic features of the TPE strains Samoa D, CDC-2 and Gauthier are shown in Table 2. All three genomes had identical GC content (52.8%) and similar length ranging between 1,139,330 bp and 1,139,744 bp. The difference of 414 bp between the largest (CDC-2) and the smallest (Samoa D) genome represents only 0.036% of the CDC-2 genome. The genome structure was identical in all strains and no major genome rearrangements were found. In all genomes, 1125 genes were annotated including 54 noncoding RNA genes. The noncoding RNA genes comprised tRNA (45), rRNA (6) and ncRNA (3) genes. The average and median gene lengths were calculated as 980 bp and 831 bp, respectively. The intergenic regions covered 53 kb in all genomes and represented 4.7% of their total genome lengths. There were 639 genes encoding proteins with predicted function in all genomes (56.8%), 140 genes encoding treponemal conserved hypothetical proteins (TCHP, i.e. proteins similar to other proteins found in the genus *Treponema*, 12.4%), 141 genes encoding conserved hypothetical proteins (CHP, 12.5%), 145 genes encoding hypothetical proteins (HP, 12.9%), and 6 annotated pseudogenes (0.6%).

**Table 2:** Summary of the genomic features of the three *T. pallidum* ssp. *pertenue* strains.

*T. pallidum* ssp. *pertenue* strains			
Genome parameter	Samoa D	CDC-2	Gauthier
Genome size	1,139,330 bp	1,139,744 bp	1,139,417 bp
G+C content	52.80%	52.80%	52.80%
No. of predicted genes	1125 including 54 untranslated genes	1125 including 54 untranslated genes	1125 including 54 untranslated genes
No. of fused genes	25 (52 corresponding genes in the Nichols genome^a^)	24 (50 corresponding genes in the Nichols genome^a^)	24 (50 corresponding genes in the Nichols genome^a^)
Sum of the intergenic region lengths (% of the genome length)	52,844 bp (4.64%)	52,963 bp (4.65%)	53,300 bp (4.68%)
Average/median gene length	980.3/831.0 bp	980.4/831.0 bp	979.3/831.0 bp
Average/median gene length of genes with unknown function	843.4/657 bp	843.8/657 bp	841.4/652.5 bp
No. of genes encoded on plus/minus DNA strand	600/525	600/525	600/525
No. of genes coding for proteins with predicted function	639	639	639
No. of genes coding for treponemal conserved hypothetical proteins	140	140	140
No. of genes coding for conserved hypothetical proteins	141	141	141
No. of genes coding for hypothetical proteins	145	145	145
No. of annotated pseudogenes (no. of all pseudogenes)	6 (13)	6 (13)	6 (13)
No. of tRNA loci	45	45	45
No. of rRNA loci	6 (2 operons)	6 (2 operons)	6 (2 operons)
No. of ncRNAs	3	3	3

a AE000520.1.

**Figure 1:**
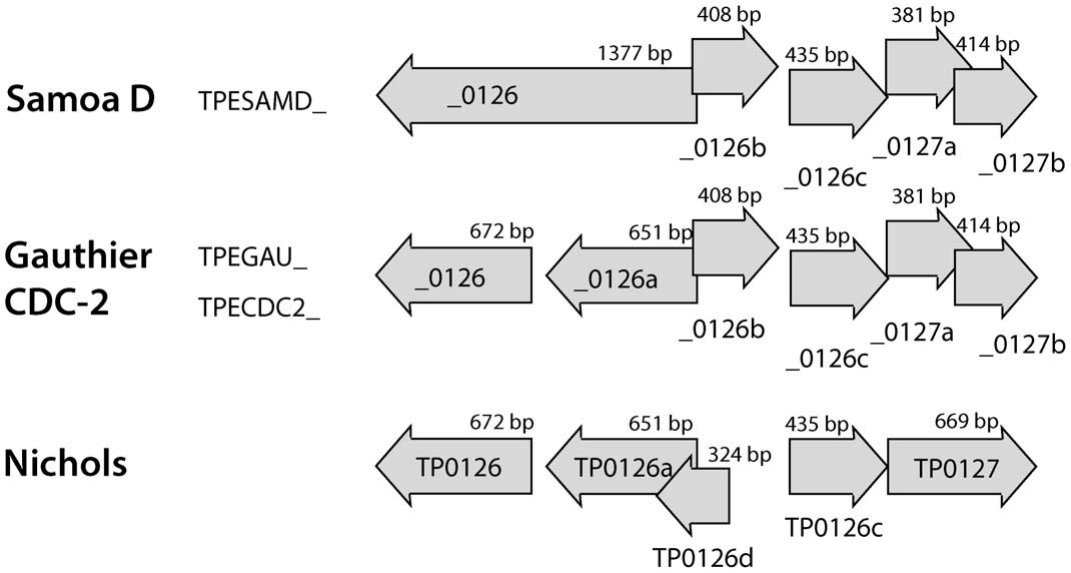
A schematic representation of the chromosomal TP0126 and TP0127 region. Newly annotated genes for each TPE strain are shown in comparison with resequenced and reannotated Nichols genes. Gene names were modified according to the GenBank instructions from the previously published ones [19]. The TP0126.1, TP0126.2, TP0126.4, and TP0126.5 genes were renamed as TP0126a, TP0126b, TP0126c and TP0126d, respectively.

With two exceptions, all genes predicted in the Samoa D genome were also predicted in the CDC-2 and Gauthier genomes. The first exception was TPESAMD_0924a (encoding a hypothetical protein), predicted only in Samoa D and not in the CDC-2 and Gauthier genomes (CDC-2 and Gauthier orthologs were shorter than the used gene length limit of 150 bp). The second exception included prediction of two genes in the CDC-2 and Gauthier genomes (TPEGAU_0126 and TPEGAU_0126a; TPECDC2_0126 and TPECDC2_0126a), while only one gene was predicted in the Samoa D genome (TPESAMD_0126). However, TPESAMD_0126a included both 0126 and 0126a sequences (Figure 1, Table 3).

**Table 3:** Gene fusions observed between TPE Samoa D genome and TPA Nichols genome.

Annotated *T. p.* ssp. *pallidum* Nichols genes (AE000520.1)	Resequenced *T. p.* ssp. *pallidum* Nichols genes^a^	Annotated *T. p*. ssp. *pertenue* Samoa D genes	Number of fused Nichols (AE000520.1) genes in the Samoa D genome
TP0006, TP0007, TP0008	TP0006_7_8	TPESAMD_0006	3
TP0013, TP0014	TP0013_14	TPESAMD_0013	2
TP0018, TP0019	TP0018_19	TPESAMD_0018	2
TP0126	TP0126, TP0126a	TPESAMD_0126^b^	2
TP0127	TP0127	TPESAMD_0127a, TPESAMD_0127b	0^c^
TP0172, TP0173	TP0172_173	TPESAMD_0172	2
TP0174, TP0175, TP0176	TP0174_175_176	TPESAMD_0174	3
TP0284, TP0285	TP0284_285	TPESAMD_0284	2
TP0286, TP0287	TP0286_287	TPESAMD_0286	2
TP0288, TP0289	TP0288_289	TPESAMD_0288	2
TP0299, TP0300	TP0299_300	TPESAMD_0300	2
TP0314, TP0315	TP0314, TP0315	TPESAMD_0314	2
TP0324, TP0325	TP0324_325	TPESAMD_0324	2
TP0377, TP0378	TP0377_378	TPESAMD_0377	2
TP0419, TP0420	TP0419_420	TPESAMD_0419	2
TP0433, TP0434	TP0433_434	TPESAMD_0433	2
TP0462, TP0463	TP0462_463	TPESAMD_0462	2
TP0468, TP0469	TP0468_469	TPESAMD_0468	2
TP0481, TP0482	TP0481_482	TPESAMD_0481	2
TP0587, TP0588	TP0587_588	TPESAMD_0587	2
TP0597, TP0598	TP0597_598	TPESAMD_0597	2
TP0702, TP0703	TP0702_703	TPESAMD_0702	2
TP0781, TP0782	TP0781_782	TPESAMD_0781	2
TP0859, TP0860	TP0859, TP0860	TPESAMD_0859	2
TP0899, TP0900	TP0899_900	TPESAMD_0899	2
TP0928, TP0929	TP0928_929	TPESAMD_0928	2

a Resequencing of selected Nichols genes (unpublished results) resulted in similar fusions (with the exceptions of TP0126, TP0126a; TP0314, TP0315; and TP0859, TP0860) as in the Samoa D genome. Gene names with underscored dash indicate fusions.

b Genes are fused only in the Samoa D and SS14 genomes, not in other *pertenue* or *pallidum* tested genomes.

c Genes are fused only in the Nichols genome and separated in all further 6 investigated genomes (Samoa D, CDC-2, Gauthier, SS14, Chicago, DAL-1).

Compared to the previously published TPA Nichols genome sequence (AE000520, [23]), the orthologs of TPE genes TP0206a, TP0451a, TP0949a, and TP0950a were later annotated in the Nichols genome (GenBank AE000520.1). These genes are therefore not listed in Table S1, which lists only newly annotated genes. Moreover, the updated Nichols genome sequence (GenBank AE000520.1) does not contain TP0950 and therefore, this gene is not shown in Table S2 (TPA Nichols genes not predicted in TPE genomes).

Compared to the Nichols genome (AE000520.1), we found 95 newly predicted genes (Table S1) in *pertenue* genomes. They coded for hypothetical or hypothetical membrane proteins (HP or HMP, 89 genes), for conserved hypothetical proteins (CHP, 3 genes), for treponemal conserved hypothetical proteins (TCHP, 2 genes) and for a transport protein (1 gene, predicted preprotein translocase). The newly annotated genes had considerably lower median length (228 bp) compared to all annotated genes (831 bp). TPA sequences orthologous to 5 of the newly predicted TPE genes (TPE_0126b, TPE_0134a, TPE_0349a, TPE_0548a, TPE_0911a) contained frameshifts or start codon mutations and were considered pseudogenes in TPA genomes.

A set of 33 genes (with a median length of 126 bp) predicted in the Nichols chromosome [23] were not annotated in TPE genomes (Table S2) because of the 150 bp gene limit (see Materials and Methods) or other genes annotated at those loci. These genes were annotated in TPA Nichols, but only as hypothetical proteins, having no similarities to genes in other genomes. However, all the corresponding orthologous sequences were present in the TPE genomes.

### Whole genome fingerprinting

The recently published data on whole genome fingerprinting of TPE genomes [19] were used to assess the overall assembly of the genome sequence and also to compare *in silico* identified restriction target sites (RTS) with experimental restriction digest patterns of individual TPI regions covering the entire TPE genomes. Altogether, 1,773 RTSs representing more than 10.6 kb were experimentally tested [19]. According to results obtained from WGF of the CDC-2 strain, one RTS (*Sph* I site, in the interval TPI21A) was missing in the complete sequence of the CDC-2 genome. Extensive sequencing of this region revealed no *Sph* I site suggesting that non-specific cleavage of the CDC-2-amplified DNA had occurred. Shorter incubations with *Sph* I (1 hour) did not result in such cleavage (data not shown). No discrepancies between *in silico* and experimental RTS analyses of Samoa D and Gauthier genomes were found. The estimated sequencing error rate for TPE genomes was therefore 10^−4^ or lower [33].

### Gene fusions

Compared to the Nichols genome (AE000520.1), 50 TPE orthologs were fused into 24 genes (see Table 3). With the exception of 2 gene fusions (TPE_0314, fusion of TP0314 and TP0315; TPE_0859, fusion of TP0859 and TP0860), all fusions found in all TPE genomes were also present in the resequenced Nichols genome (data not shown). Interestingly, all other investigated TPA strains (SS14; P. Pospíšilová, personal communication, DAL-1; data not shown; Chicago, [44]) contained the same number of fused genes as the resequenced Nichols genome including separated TP0314, TP0315, TP0859, and TP0860 genes.

### Pseudogenes

Altogether, 6 annotated and 7 non-annotated pseudogenes were found in the TPE genomes. Three pseudogenes (TPE_0146, TPE_0520, and TPE_0812) were annotated in TPE genomes as authentic frameshifts similar to the annotation of the Nichols genome (AE000520.1). Additional 3 pseudogenes were annotated only in the TPE genomes with gene counterparts in the Nichols genome (TPE_0129, TPE_0370, TPE_0671). TPE genome sequences corresponding to Nichols orthologs (TP0132, TP0135, TP0180, TP0266, TP0318, TP0532, and TP1030) were not annotated in TPE genomes as pseudogenes (Table S2) because of overlap with other genes (TPE_0532) or because gene length limits in the *de novo* annotations. However, all these orthologs were considered as pseudogenes in the TPE genomes.

### Genetic differences between syphilis and yaws treponemes

With the exception of non-annotated pseudogenes, the newly predicted TPE genes (90 genes; Table S1) and genes not predicted in TPE (33 genes; Table S2) but predicted in the Nichols genome represented differences in annotation algorithms and not real sequence changes since the corresponding orthologous sequences were present in both TPA and TPE genomes.

In addition to pseudogenes (13), the genetic differences were also characterized in 970 similarly annotated protein-coding genes in both TPE strains (Samoa D, CDC-2, and Gauthier) and TPA strains (Nichols, DAL-1, SS14, and Chicago). In a majority of these genes (in 794 out of 970, 81.9%), there were no differences in lengths of annotated orthologous genes. In 155 genes (16.0%), differences in the gene length between TPA and TPE genes resulted from different algorithms used for gene prediction or from sequencing errors in the published Nichols genome sequence (AE000520.1) and did not represent real sequence differences between TPE and TPA genomes. In addition, length differences in 14 genes were caused by strain specific sequence variants, specific for one of the sequenced TPE genomes. Therefore, these differences were also not considered as capable of differentiating between TPE and TPA genomes.

A set of seven TPE genes (TPE_0009, TPE_0103, TPE_0314, TPE_0316, TPE_0556, TPE_0859, TPE_1031) with different gene length showed consistent differences between TPA and TPE genomes (Tables 4, 5, Tables S4, and S5).

**Table 4:** TPE genes containing major sequence changes encoding proteins with predicted cell function.

Gene	Gene name	Gene/protein function	Functional gene group	Type of change in gene/protein^a^	Gene expression rate^b^	Remark	Z-test of Selection result (p)^g^
0009	***tprA***	Tpr protein A	Potential virulence factor	rev. fr. mut.	0.315	authentic frameshift mutation in the Nichols genome, MSC in Cuniculi A ortholog^c^ – originally detected in [72]	-
0103	***recQ***	ATP-dependent helicase RecQ	DNA replication, repair, recombination	fr. mut., gene elongation	0.394	MSC in Cuniculi A ortholog	-
0117	***tprC***	Tpr protein C	Potential virulence factor	MSC	0.737	MSC in Cuniculi A ortholog – originally detected in [72], recombination^d^ detected according to [56]	-
0131	***tprD***	Tpr protein D	Potential virulence factor	9 aa S	0.703	MSC in Cuniculi A ortholog – originally detected in [72], recombination detected according to [56]	-
0136		treponemal conserved hypothetical outer membrane protein	Virulence	MSC	5.199	antigen^e^, possible lipoprotein^f^, MSC in Cuniculi A ortholog, recombination detected	-
0316	***tprF***	Tpr protein F	Potential virulence factor	rev. fr. mut.	0.679	MSC in Cuniculi A ortholog – originally detected in [72], recombination detected	-
0326	***tp92***	outer membrane protein	Virulence	5 aa S, 5 aa D	0.628	antigen, MSC in Cuniculi A ortholog	Positive (0.048)
0433 TP0433-4	***arp***	treponemal conserved hypothetical protein	Potential virulence factor	MSC	1.390 1.985	MSC in Cuniculi A ortholog; antigen; recombination detected; positive selection according to [67]	-
0488	***mcp***	methyl-accepting chemotaxis protein	Cell processes	MSC	3.341	MSC in Cuniculi A ortholog	Positive (0.000)
0620	***tprI***	Tpr protein I	Potential virulence factor	10 aa S	0.654	MSC in Cuniculi A ortholog – originally detected in [72], recombination detected according to [56]	-
0621	***tprJ***	Tpr protein J	Potential virulence factor	MSC	0.751	MSC in Cuniculi A ortholog – originally detected in [72], recombination detected according to [56]	-
0671		Ethanolamine phosphotransferase	General metabolism	start codon mut.	0.197	pseudogene	
0897	***tprK***	Tpr protein K	Virulence	MSC	1.121	MSC in Cuniculi A ortholog – originally detected in [72]	-
1031	***tprL***	Tpr protein L	Potential virulence factor	MSC	0.757	MSC in Cuniculi A ortholog, recombination detected – originally detected in [72]	-

*T. p.* ssp. *pertenue* (TPE) genes encoding proteins with predicted cell function containing six or more amino acid changes and/or major sequence changes between all studied *T. p.* ssp. *pertenue* and all *T. p.* ssp. *pallidum* strains are shown.

a D, deletion; fr. mut., frameshift mutation; I, insertion; MSC (major sequence changes) are defined in Table 1; rev. fr. mut., reverted frameshift mutation; S, substitution.

b Gene expression rate in Nichols strain grown in rabbits. The gene expression rates were taken from [58].

c The gene was shown to contain frameshift mutations or MSC in the genome of *Treponema paraluiscuniculi* Cuniculi A [33].

d Detected recombination within the gene or previously identified recombination [56].

e The corresponding protein was identified as an antigen [54].

f The corresponding protein was identified as a lipoprotein [55].

g The selection test was calculated using the Kumar model [47] using MEGA4 [48] software.

**Table 5:** TPE genes containing major sequence changes encoding hypothetical proteins.

Gene	Protein prediction	Type of gene/protein change^a^	Gene expression rate^b^	Remark	Z-test Selection Type (p)^g^
0129	HP	nonsense mutation	0.763	pseudogene, MSC in Cuniculi A ortholog^c^	
0132	HP	deletions, fr. mut.	0.832	pseudogene, MSC in Cuniculi A ortholog	-
0135	HP	fr. mut.	1.526	pseudogene, MSC in Cuniculi A ortholog	-
0180	HP	fr. mut.	1.741	pseudogene, MSC in Cuniculi A ortholog	-
0133	TCHP	13 aa S	2.093	antigen^d^, possible lipoprotein^e^, MSC in Cuniculi A ortholog	
0134	TCHOMP	6 aa S	3.586	MSC in Cuniculi A ortholog	
0266	HP	MSC	3.213	pseudogene	-
0304	TCHP	6 aa S, 1 aa D	1.235	MSC in Cuniculi A ortholog	
0314 TP0314-5	TCHP	MSC, fr. mut. resulting in gene fusion	0.906 0.919	MSC in Cuniculi Aortholog, antigen	-
0318	HP	fr. mut.	0.947	pseudogene, MSC in Cuniculi A ortholog	-
0370	HP	nonsense mutation	0.822	pseudogene, MSC in Cuniculi A ortholog	
0462 TP0462-3	CHP	MSC	4.894 4.184	MSC in Cuniculi A ortholog, possible lipoprotein, antigen	Positive (0.000)
0483	TCHP	10 aa S	0.446	-	
0577	TCHMP	3 aa S, 4 aa I	0.531	MSC in Cuniculi A ortholog	
0619	TCHP	MSC	1.068	MSC in Cuniculi A ortholog, recombination detected^f^	-
0733	TCHP	6 aa S	1.735	-	
0858	TCHP	MSC	11.770	antigen, MSC in Cuniculi A ortholog	
0865	TCHOMP	11 aa S, 1 aa I	0.371	MSC in Cuniculi A ortholog	Positive (0.013)
0968	TCHP	11 aa S	3.256	MSC in Cuniculi A ortholog	Positive (0.035)
1030	HP	fr. mut.	1.46	pseudogene	-

*T. p.* ssp. *pertenue* (TPE) genes encoding hypothetical proteins containing six or more amino acid changes and/or major sequence changes between all studied *T. p.* ssp. *pertenue* and all *T. p.* ssp. *pallidum* strains are shown. CHP, conserved hypothetical protein; HP, hypothetical protein; TCHMP, treponemal conserved hypothetical membrane protein; TCHOMP, treponemal conserved hypothetical outer membrane protein; TCHP, treponemal conserved hypothetical protein.

a D, deletion; fr. mut., frameshift mutation; I, insertion; MSC (major sequence changes) are defined in Table 1; S, substitution.

b Gene expression rate in Nichols strain grown in rabbits. The gene expression rates were taken from [58].

c The gene was shown to contain frameshift mutations or MSC in the genome of *Treponema paraluiscuniculi* Cuniculi A [33].

d The corresponding protein was identified as an antigen [54].

e The corresponding protein was identified as a lipoprotein [55].

f Detected recombination.

g The selection test was calculated using the Kumar model [47] using MEGA4 [48] software.

A set of 692 out of 983 TPE (970 similarly annotated protein-coding genes in both subspecies and the 13 pseudogenes) protein encoding genes (70.4%) were found to encode either identical proteins or identical proteins with strain specific changes (Table 1, Table S3), 194 (19.7%) genes encoded proteins with 1 amino acid substitution consistently present between all tested TPE and all tested TPA genomes, 63 (6.4%) genes encoded proteins with 2 to 5 amino acid substitutions and 34 (3.5%) genes encoded proteins with 6 or more amino acid replacements, coded for full length proteins (as a result of reverted frameshift mutations), or truncated proteins (encoded by pseudogenes). TPE genes in these categories were further subclassified according to their functional groups including general metabolism, cell processes and structure, DNA metabolism, regulation and gene expression, transport, virulence and unknown function (Table 1). A statistically significant decrease was found in the category of genes encoding identical proteins in the virulence group compared to corresponding genes from the general metabolism group. A statistically significant increase was found within the virulence and unknown function groups in the category of genes encoding proteins with 6 or more amino acid replacements and/or MSC (major sequence changes, see Table 1 and Materials and Methods section for details).

### TPE genes encoding proteins with no or one amino acid replacement compared to TPA proteins

A set of 451 (45.9%) TPE genes were found to be identical in all studied TPE and TPA strains. Compared to TPA genes, 602 (61.2%) TPE genes encoded identical proteins, and 692 (70.4%) TPE genes encoded either identical proteins or identical proteins with only strain specific changes (Table S3). Altogether, 886 (90.1%) TPE genes encoded proteins with a maximum of 1 amino acid difference or strain specific changes. 283 out of these 886 genes encoded proteins of unknown function and the remaining 605 genes encoded proteins involved in the gene regulation, transcription and translation (169 genes), general metabolism (151 genes), transport (102 genes), cell processes and structure (119 genes), DNA replication, repair and recombination (46 genes), or virulence (18 genes) (see Table 1).

### Genes encoding two or more amino acid changes and/or other major sequence changes (MSC)

A set of 91 (9.3%) annotated TPE genes encoded proteins with 2 or more amino acid replacements and/or other major sequence changes. In addition to annotated TPE genes of this set, 6 TPE pseudogenes were not annotated in TPE genomes (other non-annotated pseudogene, TPE_0532, is a pseudogene also in TPA strains, see above). Forty-seven out of 97 (48.5%) genes (or pseudogenes) had no predicted function, while 50 (51.5%) of them were genes with predicted cellular function (Table 4, Table S4). Out of 50 genes with predicted function, 5 genes encoded proteins involved in DNA processing, 11 genes coded for transport proteins, 10 genes for general metabolic functions, 6 genes for cell processes and structure, 4 genes for gene regulation, transcription and translation, and 14 genes for bacterial virulence factors or potential virulence factors. The 47 predicted TPE genes (or pseudogenes) of unknown cellular function encoding proteins with 2 or more amino acid changes and/or MSC are shown in Table 5 and Table S5. Out of these 47 genes, 18 (38.3%) encoded signal peptides (data not shown), 12 of these genes encoded either membrane (8 proteins) or outer membrane proteins (4 proteins). 31 of these 47 predicted proteins were shown to interact with other treponemal proteins [53], 8 proteins were identified as antigens [54] and 2 as lipoproteins [55].

### Type of selection in divergent genes

TPE genes encoding proteins with 2 or more amino acid replacements and/or other major sequence changes were tested for prediction of selection type. Possible recombination events were identified in TPE genes orthologous to TP0136, TP0316, TP0433, TP0618, TP0619, TP0898, TP1031 and together with previously predicted recombined genes (TP0117, TP0131, TP0620, TP0621, [56]) were omitted from further analyses. All TPE orthologous genes with frameshift (TP0103, TP0132, TP0135, TP0180, TP0314, TP0318, TP1030), reverted frameshift mutations (TP0009) and long deletions (TP0266) were also excluded from analyses. In addition, the *tprK* gene, showing intrastrain variability [57], was also omitted.

In 17 and 1 of 78 tested genes, positive and purifying selection between TPA and TPE orthologous genes were identified, respectively. Nine genes with prediction of positive selection type encoded (Table 4, Table S4) proteins with predicted functions including antigens (*tp92*), proteins involved in cell processes and structure (*mcp*, *tig*, *fliK*), transport (TPE_0143, *nptA*), general metabolism (*asnA*, *thiI*) and translation (*argS*). With exceptions of *asnA*, *thiI* and *argS*, all other genes encode proteins predicted as membrane or periplasmic proteins. Eight genes (Table 5, Table S5) of unknown function were predicted under positive selection and in four of them, a signal sequence was predicted. In addition, TPE_0515 was predicted as an outer membrane protein. Moreover, 6 out these 8 hypothetical genes were highly transcribed during experimental Nichols infections in rabbits [58].

### Genetic variability among and between TPE and TPA strains

In intergenic regions, the three sequenced TPE strains differed in 16 changes (8 indels, 7 substitutions, and 1 reverse translocation of 2 rRNA loci). In the TPE coding sequences, nucleotide changes were found in 105 genes including 12 genes (TPE_0433, TPE_0470, TPESAMD_0067, TPESAMD_0326, TPESAMD_0967, TPECDC2_0136, TPECDC2_0322, TPEGAU_0131, TPEGAU_0259, TPEGAU_0629, and TPEGAU_0856a and TPEGAU_0858) with several accumulated nucleotide changes. Nucleotide changes in these 105 genes resulted in 44 synonymous, 8 conservative, 103 non-conservative amino acid substitutions, and one protein elongation (the result of a single read-through stop codon mutation). The number of relatively large specific changes (Table 6, S6) ranged from 4 in CDC-2 genome to 6 in the Gauthier genome and included genes encoding mostly outer membrane proteins, i.e. TPE_0136 (fibronectin-binding protein), TPE_0548 (treponemal conserved hypothetical membrane protein), TPE_0326 (*tp92* gene encoding outer membrane protein), TPE_0488 (*mcp* gene encoding methyl-accepting chemotaxis protein), TPE_0858 (gene encoding TCHP) and TPE_0865 (gene encoding treponemal conserved hypothetical outer membrane protein, TCHOMP).

**Table 6:** Genetic changes specific for individual TPE strain compared to the other two sequenced TPE strains.

Strain	Affected gene (predicted function)^a^	Detected strain specific change	Coordinates in AE000520	Coordinates in the Samoa D genome
**Samoa D** b	TPESAMD_0067 (unknown; CHP)	303 bp deletion	73404–73720	73402–73415
	TPESAMD_0326 (unknown; OMP)	3 bp insertion+2 nt changes	346396–346403	347762–347772
	TPESAMD_0433 (*arp* gene)	contains 12 repetitions of a 60 bp region	461078–461508	462429–463158
	TPESAMD_0470 (unknown; CHP)	contains 12 repetitions of a 24 bp region	497264–497691	498894–499201
	TPESAMD_0967 (unknown; TCHMP)	18 bp deletion	1050284–1050311	1051983–1052004
	other changes throughout the genome	4 single nt indels, 3 nt changes in intergenic regions, 60 SNPs in coding regions		
**CDC-2** b	TPECDC2_0136 (virulence; TCHOMP)	33 bp insertion	157339–157459	158172–158229
	TPECDC2_0322 (transport)	1 bp insertion and 1 bp deletion resulting in different reading frame in 32 bp region	338786–338817	340151–340182
	TPECDC2_0433 (*arp* gene)	contains 4 repetitions of a 60 bp region	461078–461508	462429–463158
	TPECDC2_0470 (unknown; CHP)	contains 37 repetitions of a 24 bp region	497264–497691	498894–499201
	other changes throughout the genome	1 single nt intergenic indel, 3 nt changes in intergenic regions, 42 nt changes in coding regions		
**Gauthier**	TPEGAU_0131 (*tprD*)	multiple indels and substitutions	151101–152897	152044–153834
	TPEGAU_0259 (unknown; CHP)	9 bp deletion	270349–270366	271116–271133
	TPEGAU_0433 (*arp* gene)	contains 10 repetitions of a 60 bp region	461078–461508	462429–463158
	TPEGAU_0470 (unknown; CHP)	contains 25 repetitions of 24 a bp region	497264–497691	498894–499201
	TPEGAU_0629 (unknown; TCHP)	302 bp deletion	686989–687300	688575–688886
	TPEGAU_0858 (unknown; TCHP) or 0856a (unknown; HP)	79 bp deletion	935475–935571	937067–937163
	other changes throughout the genome	9 single nt indels, 1 nt change in intergenic regions, 58 nt changes in coding regions		

a CHP, conserved hypothetical protein; HP, hypothetical protein; OMP, outer membrane protein; TCHMP, treponemal conserved hypothetical membrane protein; TCHOMP, treponemal conserved hypothetical outer membrane protein; TCHP, treponemal conserved hypothetical protein.

b *tprD* sequence was not included in the analysis.

TPA strains differ completely in intergenic regions with 43 changes (16 indels, 27 substitutions) and in coding regions with 379 nucleotide changes causing 96 synonymous, 33 conservative, 249 non-conservative amino acid substitution, and one protein elongation (as a consequence of a single read-through stop codon mutation).

Nucleotide diversity calculated among and between subspecies strains is summarized in Table 7. The computed average number of nucleotide differences between both subspecies was 2111.7 nucleotides, which represents 0.185% of the smallest Samoa D genome. The whole genome nucleotide divergence (*d_A_*) between subspecies is 4.7 and 4.8 times higher when compared to observed nucleotide diversity (π) within TPA and TPE strains, respectively.

**Table 7:** Calculated nucleotide diversity and divergence among and between TPA and TPE strains and subspecies.

Parameter	*T. p.* ssp. *pallidum* (TPA) strains	*T. p.* ssp. *pertenue* (TPE) strains
No. of nucleotide changes in the intergenic regions	27 (5.1 nt per 10,000 bp)	7 (1.3 nt per 10,000 bp)
No. of nucleotide changes in the coding regions^a^	379 (35 nt per 100,000 bp)	156 (14 nt per 100,000 bp)
Whole genome nucleotide diversity (π) among subspecies strains ± standard deviation^b^	0.00033±0.00015	0.00032±0.00011
Average number of nucleotide differences between subspecies strains^b^	2111.7	
Whole genome nucleotide divergence between subspecies (*d_A_*) ± standard deviation^b^	0.00154±0.00065	

a highly divergent *tprD* and *tprK* were excluded from the analysis.

b calculated from the complete genome sequences.

## Discussion

The complete genome sequences of three yaws-causing *T. pallidum* ssp. *pertenue* strains, i.e. Samoa D, CDC-2, and Gauthier, were determined. Each genome was sequenced using at least two independent techniques and the discrepancies were resequenced with Sanger sequencing to obtain high quality genome sequences. Genetic heterogeneities discovered during analysis of individual Solexa reads were, with the exception of the TPE_0110, found in the genes belonging to paralogous families. Therefore, most of the observed intrastrain heterogeneities represent rather misaligned Solexa reads than real intrastrain genetic variability. The whole genome fingerprinting data, published earlier [19], revealed a high quality sequenced genomes with assessed sequencing error rates better than 10^−4^. Deciphering the whole genome sequence of these uncultivable pathogens is an essential step before additional studies can be performed [58]–[62].

Striking sequence similarities (higher than 99.8%) to TPA genomes were found comprising identical genome structure and similar genome sizes. The Samoa D strain was isolated in Western Samoa in 1953 [63], the CDC-2 in Akorabo, Ghana in 1980 [64], and Gauthier strain in Brazzaville, Congo in 1960 [65]. Although the sequenced TPE strains were isolated at different time points and different geographical regions, their genome sizes were very similar, representing only a 414 bp difference between the largest (CDC-2) and the smallest (Samoa D) genome (0.036% of the CDC-2 genome). In addition, G+C content, number of predicted genes and gene order was identical in all genomes reflecting low nucleotide diversity of individual genomes among TPE subspecies strains (0.00032). The whole genome nucleotide divergence between TPA and TPE subspecies was 4.7 and 4.8 times higher than the observed nucleotide diversity among each subspecies indicating close evolutionary relatedness between yaws and syphilis strains. High sequence relatedness is known for other obligatory pathogenic bacteria with very restricted ecological niches [66]. The classification of syphilis and yaws treponemes into TPA and TPE subspecies, respectively, is therefore based on very subtle genetic differences. Interestingly, the yaws treponemes were found to be closely related to the unclassified treponemal simian isolate Fribourg-Blanc [19], [67]. The Fribourg-Blanc treponemes were isolated in 1962 [68] from a baboon (*Papio cynocephalus*) in Guinea, Africa. Indeed, experimental inoculation of humans with the Fribourg-Blanc strain resulted in symptoms similar to yaws [69].

**Figure 2:**
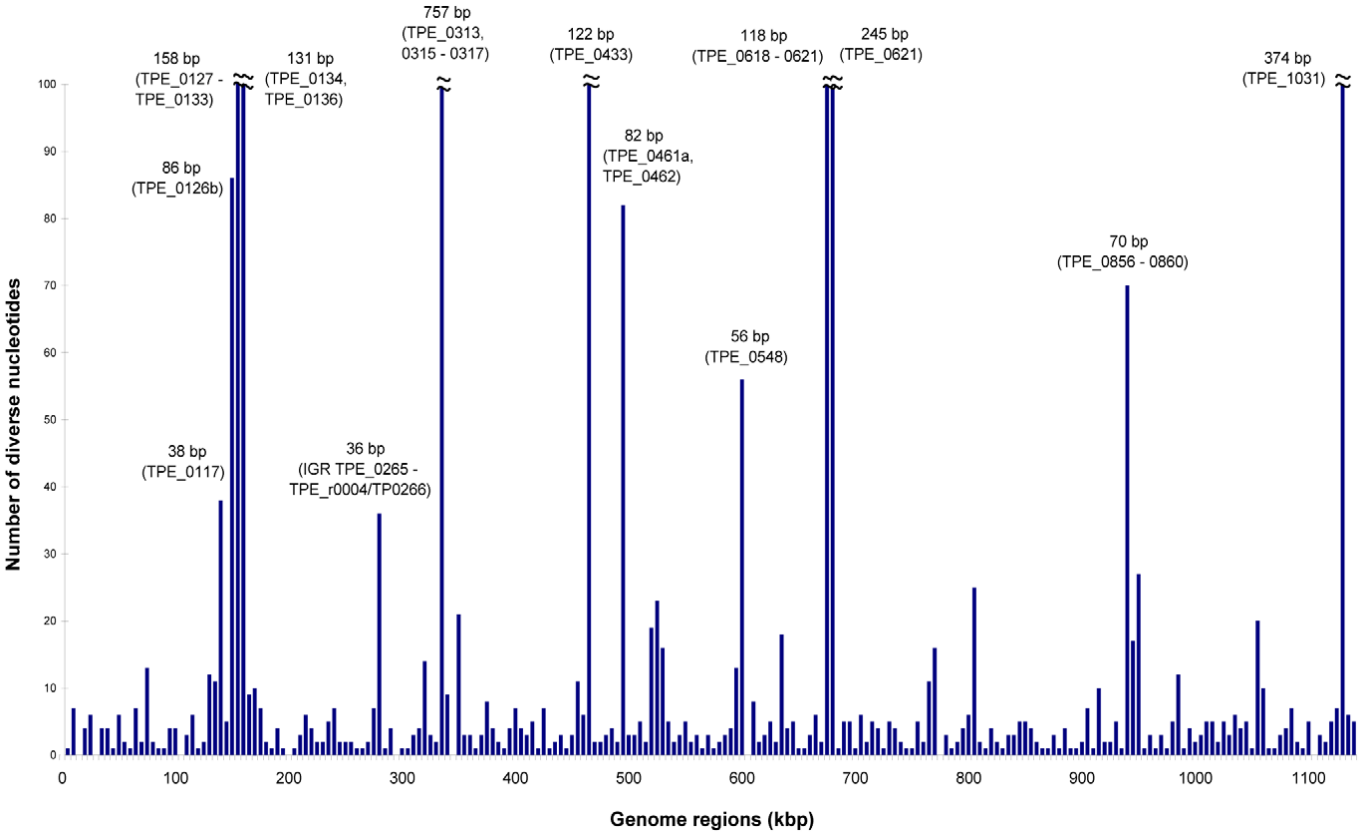
Number of nucleotide differences consistently found between all investigated *pertenue* and *pallidum* genomes. Differences shown in 5 kb-long intervals along the treponemal chromosome. Please note that the numbers of nucleotide changes (shown in bp) are for 7 intervals artificially terminated at 100 nt; the real values are shown next to corresponding vertical columns.

Although syphilis and yaws share a very similar multistage course of symptoms including a late-stage disease phase and the same histopathological changes, there are differences in clinical manifestations and epidemiology between yaws and syphilis. These differences comprise a different route of transmission, the extent of tissues and organs that can be affected during infection and transplacental infection of the fetus in syphilis [70]. In general, yaws treponemes can be considered less virulent compared to syphilis treponemes. Based on these differences, syphilis and yaws treponemes were originally considered as separate species; however, since 1984 they have been classified as subspecies [11] based on DNA hybridizations experiments [10]. Since that time, several publications showing genetic differences between TPA and TPE have been published, including differences in the TP1038 gene at position 122 [12], [13], in the 16S rRNA gene (unpublished data, cited in [14]), in the 5′- and 3′-flanking regions of TP0171 [15], in the *gpd* gene (TP0257) at position 579 [16], in tp92 [17], and in *tprI* and *tprC* [18]. In this study, about 1192 single nucleotide substitutions and 12 chromosomal regions with more extensive changes (TPE_0117, TPE_0131, TPE_0132, TPE_0136, TPE_0316, TPE_0317, TPE_0433, TPE_0548, TPE_0620, TPE_0621, TPE_0897, TPE_1031) were found to be present between all investigated TPE and TPA strains (Figure 2). This set of genetic differences represents the pool of potentially important changes differentiating syphilis and yaws treponemes. Sequencing of additional TPE and TPA genomes will eventually lead to further downsizing of this list of differences. Assuming that changes that lead to the differing pathogenic potential between yaws and syphilis treponemes represent changes in protein sequence and/or in intergenic regulatory regions (possibly affecting gene expression patterns), then the list can be shortened by 369 synonymous mutations leaving 823 intergenic and non-synonymous mutations.

Since 70.4% of genes encode either identical proteins or identical proteins with strain specific changes, potential “yaws specific” mutations should be found among 34 (3.5%) genes encoding proteins with 6 or more amino acid substitutions and/or major sequence changes (including pseudogenes), among 63 (6.4%) genes encoding proteins with 2–5 amino acid changes, or among 194 (19.7%) genes encoding proteins with 1 amino acid substitution. Since genes encoding putative virulence factors were enriched in the category of genes encoding proteins with 6 or more amino acid replacements and/or MSC compared to other gene categories (i.e. genes encoding identical proteins, genes encoding proteins with one amino acid replacement, and genes encoding proteins with 2–5 amino acid changes), it is likely that the “yaws specific” changes are to be found in the group of putative virulence factors. Similarly, an increased (though not statistically significant) percentage of genes encoding 6 or more amino acid replacements and/or MSC was found in the group of genes of unknown function. In fact, some of the hypothetical proteins may encode for yet unknown virulence factors. This prediction is supported by the fact that the genes encoding hypothetical proteins with 6 or more amino acid replacements and/or MSC (20 genes, Table 5) are highly transcribed (average transcription rate 2.3) in the TPA Nichols strain during experimental rabbit infection [58], while the average transcription rate calculated from values for all genes was 1.0. In addition to this finding, several of these genes were found to contain MSC in the genome of non-human pathogen *T. paraluiscuniculi* Cuniculi A [33], further suggesting their potential role in human treponemal infections. Moreover, genes responsible for “yaws specific” changes could be found in the group of 17 genes predicted under positive selection between TPA and TPE strains. The positively selected genes were often found to encode proteins with predicted signal sequences or membrane proteins including predicted lipoproteins and outer membrane proteins suggesting that these proteins may be responsible for differences in pathogenesis between syphilis and yaws. Genes with predicted positive selection encoding components involved in general metabolism, transport, cell processes and cell structure suggest that positive selection of these genes may be related to adaptive processes, such as climate adaptation. As shown by previous studies [15], [56], [67], *T. pallidum* ssp. *endemicum* is very closely related to *pertenue* subspecies. Therefore, many “yaws specific” mutations are likely to be in the future identified also in the ssp. *endemicum* strains. For instance, out of 1192 nucleotide substitutions differentiating all tested TPE strains from all TPA strains, 551 (46%) were found also in the *T. paraluiscuniculi* genome.

The only pseudogene with a known function (TPE_0671), identified in the TPE genomes, coded for ethanolamine phosphotransferase. The phosphoethanolamine transferase of *Campylobacter jejuni* is involved in modification of the lipooligosaccharide lipid anchor lipid A and also in the modification of the flagellar rod protein FlgG [71]. Although the role of this enzyme is not known in lipopolysaccharide-free treponemes, the *flgG* genes (TP0960, TP0961) are present and conserved in the TPA and TPE genomes. On the other hand, a low expression rate of TP0671, in TPA Nichols [58], may suggest a nonessential role for this gene.

In addition to the ethanolamine phosphotransferase pseudogene (TPE_0671), 13 additional genes with predicted function showed 6 or more amino acid changes and/or MSC including 8 *tpr* genes. Although the function of Tpr proteins were not directly demonstrated, the Tpr proteins are considered important factors in pathogenesis and/or immune evasion [56], [72]. Tpr proteins were shown to exhibit heterogeneity both among and between the *T. pallidum* subspecies and strains and were shown to induce an antibody response during treponemal infection [73]–[75]. Moreover, a gene conversion-driven model for antigenic variations of TprK during experimental infection has been proposed [76]. This work provides further indirect evidence of possible roles of *tpr* genes in treponemal virulence. Interestingly, eight *tpr* genes including *tprB,C,D,E,F,I,J,L* out of 12 *tpr* genes were predicted to be genes encoding rare outer membrane proteins (OMPs) in *T. pallidum* [77]. Since six out of these 8 OMPs were found to contain changes in TPE strains, the Tpr rare OMPs may contribute to differences in pathogenesis between syphilis and yaws. Besides the elongated RecQ protein (TPE_0103) and sequentially divergent Mcp (methyl-accepting chemotaxis protein, TPE_0488), three treponemal antigens were found to display substantial sequence diversity: the fibronectin-binding protein (TPE_0136, [78]), the Tp92 – outer membrane protein (TPE_0326) and the Arp protein (TPE_0433).

TP0136 is an outer membrane protein involved in attachment to host extracellular matrix proteins. Although immunization with recombinant TP0136 did not prevent TPA infection, delayed ulceration in experimental animals has been demonstrated [78]. The Tp92 is an outer membrane antigen that partially protects rabbits from subsequent TPA infection [17]. Tests with recombinant Tp92 protein exhibited high sensitivity and specificity values and Tp92 is considered a promising diagnostic antigen in syphilis serodiagnostics [79]. Moreover, tests of Tp92 homologs, that are common among the oral *Treponema* species associated with periodontitis, have shown that these proteins have the potential to bind to host cells and stimulate host factors that contribute to inflammation and osteoclastogenesis [80]. The *arp* gene [81] contains a variable number of 60 bp-repetitive sequences and corresponds to the TP0433 and TP0434 gene loci of the published Nichols genome sequence [23]. The Arp protein has been shown to be immunogenic [82] and the variable number of its tandem repeats suggests important biological function [83], [84]. Based on amino acid sequence variations, Arp repeat motifs were classified into 4 types in *T. pallidum* (I, II, III, II/III) [67], [82] and the variability of its repeat types was correlated with a sexual transmission strategy [67] with invariant repeat type (II) in TPE strains.

Current serological diagnostic methods do not allow discrimination between yaws and syphilis infections. Although the differences in clinical manifestation and epidemiology between yaws and syphilis allow correct diagnosis in most cases, sometimes it is quite difficult or impossible to distinguish between yaws and congenital syphilis in children [85] or in pregnant women [86]. This can lead to improper diagnosis and treatment with important health consequences [86]. A complete compendium of TPE-specific differences is presented in this work and should allow selection of the most suitable chromosomal regions for differential identification of TPE infections. The correct diagnostics of yaws treponemes is also one of important steps in monitoring and eradication of yaws. Moreover, a list of specific changes for each of the investigated TPE genomes (Samoa D, CDC-2, and Gauthier) was also generated. Although these changes cannot account for differences in pathogenicity between TPA and TPE, they represent suitable targets for specific molecular diagnostics of individual strains.

Taken together, the yaws treponemes are very closely related to syphilis treponemes although they are less virulent. TPE genomes differ minimally from TPA genomes with approximately 5-times higher nucleotide divergence than the nucleotide diversity observed among TPA and TPE subspecies strains, respectively. The decreased degree of TPE virulence is likely the result of accumulation of genetic changes in *tpr* genes, genes encoding prominent antigens, and some other genes. Genes coding for hypothetical proteins with consistently identified nucleotide differences between TPE and TPA strains are candidates for virulence factors of syphilitic treponemes.

## Supporting Information

Table S1 **Newly predicted genes in** ***T. p.*** **ssp.** ***pertenue*** **.** A set of 95 genes newly predicted in the *T. p.* ssp. *pertenue* (TPE) genomes when compared to *T. p.* ssp. *pallidum* (TPA) Nichols genome annotation (AE000520.1).
(DOC)

Table S2 ***T. p.*** **ssp.** ***pallidum*** **Nichols genes not annotated in** ***T. p.*** **ssp.** ***pertenue*** **genomes.** Genes predicted in the Nichols chromosome were not annotated in TPE genomes because of the 150 bp gene limit or other genes annotated at those loci. However, all the corresponding orthologous sequences were present in the TPE genomes.
(DOC)

Table S3 **Genes encoding identical proteins in all** ***T. p.*** **ssp.** ***pertenue*** **and all** ***T. p.*** **ssp.** ***pallidum*** **strains.**
(DOC)

Table S4 ***T. p.*** **ssp.** ***pertenue*** **genes containing major sequence changes encoding proteins with predicted cell function.** *T. p.* ssp. *pertenue* genes encoding proteins with predicted cell function containing two to five amino acid changes when compared to *T. p.* ssp. *pallidum* genes.
(DOC)

Table S5 ***T. p.*** **ssp.** ***pertenue*** **genes containing major sequence changes encoding hypothetical proteins.** *T. p.* ssp. *pertenue* genes encoding proteins with unknown cell function containing two to five amino acid changes when compared to *T. p.* ssp. *pallidum* genes.
(DOC)

Table S6 **Genetic variability among TPE (CDC-2, Samoa D and Gauthier) strains genomes.**
(XLS)
