# Supplementary material for: Whole Genome Sequences of Three Treponema pallidum ssp. pertenue Strains: Yaws and Syphilis Treponemes Differ in Less than 0.2% of the Genome Sequence
Source: PLoS Negl Trop Dis. 2012 Jan 24;6(1):e1471. doi: 10.1371/journal.pntd.0001471 (PMC3265458; doi:10.1371/journal.pntd.0001471)
Supplement: Table S1 — Newly predicted genes in T. p. ssp. pertenue . A set of 95 genes newly predicted in the T. p. ssp. pertenue (TPE) genomes when compared to T. p. ssp. pallidum (TPA) Nichols genome annotation (AE000520.1). (DOC) [file pntd.0001471.s001.doc]

***Table S1. A set of 95 genes newly predicted in the Treponema pallidum ssp. pertenue (TPE) genomes when compared to T. p. ssp. pallidum (TPA) Nichols genome annotation (AE000520.1)***

| **Predicted gene** | **DNA strand** | **Gene/protein function** | **Protein length (aa)** |
| --- | --- | --- | --- |
|
| TPE_0001a | - | HP | 76 |
| TPE_0005a | + | HP | 86 |
| TPE_0045a | + | HP | 89 |
| TPE_0054a | + | HP | 57 |
| TPE_0063a | - | HP | 93 |
| TPE_0067a | + | HP | 91 |
| TPE_0073a | - | HP | 64 |
| TPE_0082a | + | HP | 100 |
| TPE_0118a | + | HP | 51 |
| TPESAMD_0126 TPECDC2_0126a TPEGAU_0126a | - | HP | 458  216  216 |
| TPE_0126b | + | HP | 135 |
| TPE_0126c | + | HP | 144 |
| TPE_0129a | - | HP | 142 |
| TPE_0129b | + | HP | 113 |
| TPE_0134a | + | HP | 210 |
| TPE_0134b | + | TCHP | 235 |
| TPE_0136a | + | HP | 63 |
| TPE_0140a | - | HP | 56 |
| TPE_0152a | - | HP | 85 |
| TPE_0163a | + | HP | 71 |
| TPE_0217a | + | HP | 51 |
| TPE_0226a | + | HP | 52 |
| TPE_0240a | + | HP | 49 |
| TPE_0258a | + | HP | 49 |
| TPE_0264a | - | HP | 58 |
| TPE_0274a | + | HP | 58 |
| TPE_0312a | + | HP | 53 |
| TPE_0328a | - | HP | 52 |
| TPE_0344a | - | HP | 96 |
| TPE_0345a | + | HP | 66 |
| TPE_0349a | + | HP | 50 |
| TPE_0353a | - | HP | 126 |
| TPE_0359a | - | HP | 83 |
| TPE_0361a | - | HP | 50 |
| TPE_0362a | - | HMP | 138 |
| TPE_0366a | - | HP | 113 |
| TPE_0374a | + | HP | 86 |
| TPE_0374b | - | CHP | 98 |
| TPE_0409a | + | preprotein translocase subunit YajC | 125 |
| TPE_0411a | + | HP | 53 |
| TPE_0435a | - | HP | 55 |
| TPE_0453a | + | CHP | 116 |
| TPE_0461a | - | HP | 60 |
| TPE_0494a | + | HP | 62 |
| TPE_0505a | + | HP | 88 |
| TPE_0525a | - | HP | 50 |
| TPE_0536a | - | HP | 68 |
| TPE_0537a | - | HP | 58 |
| TPE_0538a | + | HP | 60 |
| TPE_0540a | - | HP | 75 |
| TPE_0542a | - | HP | 61 |
| TPE_0547a | + | HP | 70 |
| TPE_0548a | + | HP | 65 |
| TPE_0574a | - | HP | 90 |
| TPE_0591a | + | HP | 92 |
| TPE_0608a | + | HP | 55 |
| TPE_0637a | - | HP | 61 |
| TPE_0641a | - | HP | 68 |
| TPE_0669a | + | HP | 55 |
| TPE_0683a | - | HP | 81 |
| TPE_0705a | - | HP | 57 |
| TPE_0705b | - | HP | 52 |
| TPE_0736a | - | HP | 56 |
| TPE_0743a | - | HP | 52 |
| TPE_0766a | - | HP | 56 |
| TPE_0774a | - | HP | 83 |
| TPE_0775a | - | HP | 51 |
| TPE_0783a | + | HP | 56 |
| TPE_0783b | - | HP | 65 |
| TPE_0798a | - | HP | 52 |
| TPE_0798b | - | HP | 89 |
| TPE_0814a | + | HP | 71 |
| TPE_0814b | - | HP | 78 |
| TPE_0823a | - | HP | 145 |
| TPE_0841a | + | HP | 78 |
| TPE_0856a | - | HP | 456 |
| TPE_0861a | - | HP | 102 |
| TPE_0867a | - | HP | 66 |
| TPE_0908a | - | HP | 82 |
| TPE_0911a | - | HP | 91 |
| TPE_0913a | + | TCHP | 60 |
| TPE_0919a | + | HP | 52 |
| TPE_0921a | + | CHP | 350 |
| TPE_0926a | - | HP | 99 |
| TPE_0927a | - | HP | 89 |
| TPE_0933a | + | HP | 74 |
| TPE_0951a | - | HP | 92 |
| TPE_0954a | + | HP | 97 |
| TPE_0971a | - | HP | 51 |
| TPE_0980a | - | HP | 78 |
| TPE_0984a | - | HP | 104 |
| TPE_0989a | + | HP | 77 |
| TPE_0993a | - | HP | 98 |
| TPE_1017a | - | HP | 63 |
| TPE_1025a | + | HP | 140 |

HP, hypothetical protein; CHP, conserved hypothetical protein; TCHP, treponemal conserved hypothetical protein; HMP, hypothetical membrane protein.

In addition to genes shown in Table S1, TPESAMD_0924a (HP, 77 aa) was predicted in Samoa D genome and not in the CDC-2 and Gauthier genomes.
