# Supplementary material for: Whole Genome Sequences of Three Treponema pallidum ssp. pertenue Strains: Yaws and Syphilis Treponemes Differ in Less than 0.2% of the Genome Sequence
Source: PLoS Negl Trop Dis. 2012 Jan 24;6(1):e1471. doi: 10.1371/journal.pntd.0001471 (PMC3265458; doi:10.1371/journal.pntd.0001471)
Supplement: Table S2 — T. p. ssp. pallidum Nichols genes not annotated in T. p. ssp. pertenue genomes. Genes predicted in the Nichols chromosome were not annotated in TPE genomes because of the 150 bp gene limit or other genes annotated at those loci. However, all the corresponding orthologous sequences were present in the TPE genomes. (DOC) [file pntd.0001471.s002.doc]

**Table S2. *Treponema pallidum* ssp. *pallidum* Nichols (TPA) genes not annotated in *Treponema* *pallidum* ssp. *pertenue*** (TPE) genomes

| **Annotated genes in the Nichols strain (AE000520.1)^a^** | **Gene length (bp)** | **Reason for omitting the gene from annotation** | **Note** |
| --- | --- | --- | --- |
| TP0010 | 126 | length limit^b^ |  |
| TP0041 | 123 | length limit |  |
| TP0132 | 210 | frameshift mutation, length limit | pseudogene in TPE |
| TP0135 | 942 | frameshift mutation, 2 other genes were annotated at this region (TP0134a, TP0134b) | pseudogene in TPE |
| TP0137 | 138 | length limit |  |
| TP0161 | 93 | length limit |  |
| TP0169 | 99 | length limit |  |
| TP0180 | 159 | frameshift mutation, length limit | pseudogene in TPE |
| TP0224 | 105 | length limit |  |
| TP0232 | 117 | length limit |  |
| TP0266 | 126 | partial gene deletion, length limit | pseudogene in TPE |
| TP0278 | 129 | length limit |  |
| TP0280 | 129 | length limit |  |
| TP0281 | 129 | length limit |  |
| TP0311 | 144 | length limit |  |
| TP0318 | 180 | frameshift mutation, length limit | pseudogene in TPE |
| TP0332 | 126 | length limit |  |
| TP0375 | 210 | other gene was annotated at this region (TP0374a, 261 bp) |  |
| TP0382 | 123 | length limit |  |
| TP0504 | 141 | length limit |  |
| TP0532 | 467 | authentic frameshift, length limit | pseudogene in TPE |
| TP0539 | 222 | other gene was annotated at this region (TP0538a, 183 bp) |  |
| TP0573 | 93 | length limit |  |
| TP0583 | 126 | length limit |  |
| TP0590 | 117 | length limit |  |
| TP0607 | 147 | length limit |  |
| TP0656 | 102 | length limit |  |
| TP0699 | 111 | length limit |  |
| TP0723 | 135 | length limit |  |
| TP0759 | 120 | length limit |  |
| TP0799 | 153 | other gene was annotated at this region (TP0798a, 159 bp) |  |
| TP0811 | 126 | length limit |  |
| TP0857 | 321 | 2 other genes were annotated at this region (TP0856 and TP0856a) |  |
| TP0916 | 129 | length limit |  |
| TP0922 | 894 | other gene was annotated at this region (TP0921a) |  |
| TP0932 | 93 | length limit |  |
| TP0940 | 141 | length limit |  |
| TP0955 | 273 | other gene was annotated at this region (TP0954a, 294 bp) |  |
| TP0970 | 117 | length limit |  |
| TP1030 | 498 | partial gene deletion, frameshift, other gene was annotated at this region (TP1031, 1671 bp) | pseudogene in TPE |

^a^[23]

^b^Hypothetical genes shorter than 150 bp were not annotated in the TPE genomes.
