# Supplementary material for: Whole Genome Sequences of Three Treponema pallidum ssp. pertenue Strains: Yaws and Syphilis Treponemes Differ in Less than 0.2% of the Genome Sequence
Source: PLoS Negl Trop Dis. 2012 Jan 24;6(1):e1471. doi: 10.1371/journal.pntd.0001471 (PMC3265458; doi:10.1371/journal.pntd.0001471)
Supplement: Table S3 — Genes encoding identical proteins in all T. p. ssp. pertenue and all T. p. ssp. pallidum strains. (DOC) [file pntd.0001471.s003.doc]

**Table S3. 692 genes encoding identical proteins in all *T. p.* ssp. *pertenue* (TPE) and all *T. p*. ssp. *pallidum*** (TPA) strains

| **Gene** | **Gene/protein function** | **Gene name** | **Protein size (aa)** | **Functional group** |
| --- | --- | --- | --- | --- |
| TPE_0027 | possible hemolysin HlyC |  | 457 | Cell processes |
| TPE_0028 | possible hemolysin HlyC |  | 453 | Cell processes |
| TPE_0030 | chaperone GroEL | ***groEL*** | 544 | Cell processes |
| TPE_0040 | probable methyl-accepting chemotaxis protein | ***mcp*** | 814 | Cell processes |
| TPE_0071 | S14 family endopeptidase ClpB | ***clpB*** | 878 | Cell processes |
| TPE_0072 | glutaredoxin-related protein |  | 90 | Cell processes |
| TPE_0098 | chaperone DnaJ | ***dnaJ*** | 218 | Cell processes |
| TPE_0181 | probable septum formation initiator | ***divIC*** | 147 | Cell processes |
| TPE_0185 | possible signal peptidase I | ***lepB*** | 235 | Cell processes |
| TPE_0215 | chaperone GrpE | ***grpE*** | 220 | Cell processes |
| TPE_0216 | chaperone DnaK | ***dnaK*** | 635 | Cell processes |
| TPE_0217 | chaperone DnaJ | ***dnaJ*** | 374 | Cell processes |
| TPE_0251 | bacterial DNA-binding factor |  | 105 | Cell processes |
| TPE_0277 | C-terminal processing peptidase | ***prc*** | 448 | Cell processes |
| TPE_0327 | outer membrane protein H | ***ompH*** | 172 | Cell processes |
| TPE_0349 | peptidylprolyl isomerase | ***fkpB*** | 176 | Cell processes |
| TPE_0373 | PP-loop family ATPase |  | 477 | Cell processes |
| TPE_0377 (TP0377, TP0378) | flagellar basal body-associated protein FliL | ***fliL*** | 167 | Cell processes |
| TPE_0387 | cell division protein FtsW | ***ftsW*** | 384 | Cell processes |
| TPE_0388 | cell division protein FtsQ | ***ftsQ*** | 271 | Cell processes |
| TPE_0389 | cell division protein FtsA | ***ftsA*** | 414 | Cell processes |
| TPE_0390 | cell division protein FtsZ | ***ftsZ*** | 418 | Cell processes |
| TPE_0419 (TP0419, TP0420) | acid phosphatase | ***surE*** | 256 | Cell processes |
| TPE_0497 | cell shape determining protein MreB | ***mreB*** | 344 | Cell processes |
| TPE_0498 | cell shape determining protein MreC | ***mreC*** | 287 | Cell processes |
| TPE_0499 | cell shape determining protein MreD | ***mreD*** | 168 | Cell processes |
| TPE_0500 | penicillin-binding protein | ***pbp*** | 624 | Cell processes |
| TPE_0501 | cell shape determining protein RodA | ***rodA*** | 433 | Cell processes |
| TPE_0507 | S14 family endopeptidase ClpP | ***clpP*** | 203 | Cell processes |
| TPE_0508 | S14 family endopeptidase ClpX | ***clpX*** | 415 | Cell processes |
| TPE_0524 | S16 family endopeptidase La | ***lon*** | 881 | Cell processes |
| TPE_0549 | S14 family endopeptidase ClpA | ***clpA*** | 814 | Cell processes |
| TPE_0614 | bifunctional cysteine desulfurase/ selenocysteine lyase | ***sufS*** | 404 | Cell processes |
| TPE_0630 | protein-glutamate O-methyltransferase | ***cheR*** | 303 | Cell processes |
| TPE_0640 | methyl-accepting chemotaxis protein | ***mcp*** | 614 | Cell processes |
| TPE_0649 | possible hemolysin | ***tlyC*** | 265 | Cell processes |
| TPE_0712 | possible flagellar synthesis regulator FleN | ***fleN*** | 304 | Cell processes |
| TPE_0760 | penicillin-binding protein | ***pbp*** | 652 | Cell processes |
| TPE_0801 | S14 family endopeptidase ClpA | ***clpA*** | 809 | Cell processes |
| TPE_0814 | thioredoxin-disulfide reductase | ***trxB*** | 307 | Cell processes |
| TPE_0853 | possible flagellar synthesis regulator FleN | ***fleN*** | 378 | Cell processes |
| TPE_0946 | glucose-inhibited division protein B | ***gidB*** | 222 | Cell processes |
| TPE_0978 | signal peptidase II | ***lspA*** | 197 | Cell processes |
| TPE_0999 | S-DNA-T family septal DNA translocator | ***ftsK*** | 799 | Cell processes |
| TPE_1016 | possible peptidylprolyl isomerase | ***surA*** | 361 | Cell processes |
| TPE_1037 | possible hemolysin III | ***hlyIII*** | 238 | Cell processes |
| TPE_1041 | S14 family endopeptidase ClpP | ***clpP*** | 199 | Cell processes |
| TPE_0026 | flagellar motor switch protein FliG | ***fliG*** | 340 | Cell structure |
| TPE_0077 | capsular polysaccharide biosynthesis protein CapD | ***capD*** | 538 | Cell structure |
| TPE_0247 | N-acetylmuramoyl-L-alanine amidase | ***amiC*** | 357 | Cell structure |
| TPE_0249 | flagellar filament outer layer protein FlaA | ***flaA*** | 350 | Cell structure |
| TPE_0345 | phospho-N-acetylmuramoyl-pentapeptide-transferase | ***mraY*** | 363 | Cell structure |
| TPE_0386 | UDP-N-acetylmuramoyl-tripeptide--D-alanyl-D-alanine ligase | ***murF*** | 459 | Cell structure |
| TPE_0396 | flagellar basal-body rod protein FlgB | ***flgB*** | 136 | Cell structure |
| TPE_0397 | flagellar basal body rod protein FlgC | ***flgC*** | 151 | Cell structure |
| TPE_0398 | flagellar hook-basal body protein FliE | ***fliE*** | 124 | Cell structure |
| TPE_0400 | flagellar motor switch protein FliG | ***fliG*** | 352 | Cell structure |
| TPE_0403 | flagellar protein FliJ | ***fliJ*** | 150 | Cell structure |
| TPE_0406 | glutamate racemase | ***murI*** | 268 | Cell structure |
| TPE_0417 | apolipoprotein N-acyltransferase | ***int*** | 559 | Cell structure |
| TPE_0435 | copper resistance lipoprotein NlpE | ***nlpE*** | 156 | Cell structure |
| TPE_0440 | pyridoxal phosphate-dependent transferase | ***wecE*** | 383 | Cell structure |
| TPE_0453 | treponemal conserved hypothetical membrane protein |  | 287 | Cell structure |
| TPE_0523 | undecaprenyldiphospho-muramoylpentapeptide beta-N- acetylglucosaminyltransferase | ***murG*** | 384 | Cell structure |
| TPE_0562 | N-acetylneuraminate synthase | ***spsE*** | 378 | Cell structure |
| TPE_0571 | LemA family protein |  | 221 | Cell structure |
| TPE_0658 | flagellar assembly protein FliW | ***fliW*** | 150 | Cell structure |
| TPE_0659 | flagellar hook-associated protein FlgL | ***flgL*** | 416 | Cell structure |
| TPE_0660 | flagellar hook-associated protein FlgK | ***flgK*** | 625 | Cell structure |
| TPE_0681 | alanine racemase | ***alr*** | 377 | Cell structure |
| TPE_0688 | muramoyltetrapeptide carboxypeptidase | ***ldcA*** | 337 | Cell structure |
| TPE_0705 | bifunctional membrane carboxypeptidase/penicillin-binding protein | ***mrcA*** | 884 | Cell structure |
| TPE_0713 | flagellar-associated GTP-binding protein FlhF | ***flhF*** | 437 | Cell structure |
| TPE_0719 | possible flagellar biogenesis protein FliO | ***fliO*** | 262 | Cell structure |
| TPE_0721 | flagellar motor switch protein FliM | ***fliM*** | 344 | Cell structure |
| TPE_0722 | flagellar basal body-associated protein FliL | ***fliL*** | 182 | Cell structure |
| TPE_0727 | flagellar hook protein FlgE | ***flgE*** | 463 | Cell structure |
| TPE_0728 | flagellar basal body rod modification protein FlgD | ***flgD*** | 153 | Cell structure |
| TPE_0769 | treponemal membrane protein B | ***tmpB*** | 325 | Cell structure |
| TPE_0792 | flagellar filament core protein FlaB | ***flaB*** | 286 | Cell structure |
| TPE_0806 | FemAB family protein |  | 498 | Cell structure |
| TPE_0868 | flagellar filament core protein FlaB | ***flaB*** | 286 | Cell structure |
| TPE_0870 | flagellar filament core protein FlaB | ***flaB*** | 285 | Cell structure |
| TPE_0933 | UDP-N-acetylmuramoyl-L-alanyl-D-glutamate--2,6-diaminopimelate ligase | ***murE*** | 540 | Cell structure |
| TPE_0960 | flagellar basal body rod protein FlgG | ***flgG*** | 264 | Cell structure |
| TPE_0961 | flagellar basal body rod protein FlgG | ***flgG*** | 271 | Cell structure |
| TPE_0001 | DNA-directed DNA replication initiator protein | ***dnaA*** | 464 | DNA replication, repair, recombination |
| TPE_0002 | DNA-directed DNA polymerase III beta subunit | ***dnaN*** | 371 | DNA replication, repair, recombination |
| TPE_0003 | recombination protein F | ***recF*** | 377 | DNA replication, repair, recombination |
| TPE_0058 | replicative DNA helicase DnaB | ***dnaB*** | 438 | DNA replication, repair, recombination |
| TPE_0062 | single-stranded DNA-binding protein | ***ssb*** | 176 | DNA replication, repair, recombination |
| TPE_0141 | methylated-DNA--[protein]-cysteine S-methyltransferase | ***ada*** | 177 | DNA replication, repair, recombination |
| TPE_0162 | crossover junction endoribonuclease subunit B | ***ruvB*** | 350 | DNA replication, repair, recombination |
| TPE_0229 | uracil DNA glycosylase | ***udg*** | 270 | DNA replication, repair, recombination |
| TPE_0310 | probable single-stranded DNA-binding protein | ***ssb*** | 127 | DNA replication, repair, recombination |
| TPE_0328 | DNA mismatch repair protein MutS | ***mutS*** | 900 | DNA replication, repair, recombination |
| TPE_0343 | probable A/G-specific adenine glycosylase | ***mutY*** | 277 | DNA replication, repair, recombination |
| TPE_0353 | ribonuclease H | ***rnhA*** | 169 | DNA replication, repair, recombination |
| TPE_0380 | possible DNA repair helicase |  | 606 | DNA replication, repair, recombination |
| TPE_0391 | integrase/recombinase XerD | ***xerD*** | 306 | DNA replication, repair, recombination |
| TPE_0393 | SMF family protein | ***dprA*** | 302 | DNA replication, repair, recombination |
| TPE_0442 | DNA repair protein RecN | ***recN*** | 573 | DNA replication, repair, recombination |
| TPE_0472 | excision endonuclease subunit UvrC | ***uvrC*** | 691 | DNA replication, repair, recombination |
| TPE_0514 | excision endonuclease subunit UvrA | ***uvrA*** | 960 | DNA replication, repair, recombination |
| TPE_0517 | crossover junction endoribonuclease subunit C | ***ruvC*** | 196 | DNA replication, repair, recombination |
| TPE_0526 | ATP-dependent helicase HrpA | ***hrpA*** | 668 | DNA replication, repair, recombination |
| TPE_0543 | crossover junction endoribonuclease subunit A | ***ruvA*** | 227 | DNA replication, repair, recombination |
| TPE_0587 (TP0587, TP0588) | DNA-directed DNA polymerase III delta subunit | ***holA*** | 340 | DNA replication, repair, recombination |
| TPE_0626 | exonuclease SbcD | ***sbcD*** | 391 | DNA replication, repair, recombination |
| TPE_0634 | DNA ligase (NAD(+)) | ***lig*** | 823 | DNA replication, repair, recombination |
| TPE_0636 | recombination protein O | ***recO*** | 256 | DNA replication, repair, recombination |
| TPE_0643 | DNA-directed DNA polymerase III epsilon subunit | ***dnaQ*** | 215 | DNA replication, repair, recombination |
| TPE_0669 | DNA-directed DNA polymerase III alpha subunit | ***dnaE*** | 1170 | DNA replication, repair, recombination |
| TPE_0687 | DNA helicase RecG | ***recG*** | 686 | DNA replication, repair, recombination |
| TPE_0704 | single-stranded-DNA-specific exonuclease | ***recJ*** | 706 | DNA replication, repair, recombination |
| TPE_0775 | DNA-(apurinic or apyrimidinic site) lyase | ***nth*** | 211 | DNA replication, repair, recombination |
| TPE_0810 | site-specific DNA-methyltransferase (adenine-specific) | ***dam*** | 303 | DNA replication, repair, recombination |
| TPE_1004 | recombination protein R | ***recR*** | 201 | DNA replication, repair, recombination |
| TPE_1005 | DNA-directed DNA polymerase III gamma and tau subunits | ***dnaZX*** | 572 | DNA replication, repair, recombination |
| TPE_1006 | DNA topoisomerase (ATP-hydrolyzing) subunit B | ***gyrB*** | 637 | DNA replication, repair, recombination |
| TPE_1028 | excision endonuclease subunit UvrD | ***uvrD*** | 670 | DNA replication, repair, recombination |
| TPE_0025 | M16C subfamily peptidase |  | 1023 | General metabolism |
| TPE_0037 | D-lactate dehydrogenase | ***ldhA*** | 331 | General metabolism |
| TPE_0045 | adenosine deaminase | ***add*** | 299 | General metabolism |
| TPE_0049 | M23B subfamily peptidase |  | 363 | General metabolism |
| TPE_0050 | possible phosphoribosyltransferase |  | 202 | General metabolism |
| TPE_0065 | probable SAM dependent methyltransferase |  | 181 | General metabolism |
| TPE_0068 | probable Fe-S-cluster redox enzyme |  | 340 | General metabolism |
| TPE_0078 | DegT/DnrJ/EryC1/StrS family pyridoxal dependent aminotransferase |  | 412 | General metabolism |
| TPE_0080 | xanthine dehydrogenase | ***coxS*** | 155 | General metabolism |
| TPE_0094 | phosphate acetyltransferase | ***pta*** | 336 | General metabolism |
| TPE_0099 | UMP kinase | ***pyrH*** | 251 | General metabolism |
| TPE_0100 | TlpA family thioredoxin-disulfide reductase |  | 200 | General metabolism |
| TPE_0104 | bifunctional 5'-nucleotidase/UDP-sugar diphosphatase | ***ushA*** | 593 | General metabolism |
| TPE_0108 | 6-phosphofructokinase | ***pfk*** | 461 | General metabolism |
| TPE_0112 | bleomycin hydrolase | ***pepC*** | 450 | General metabolism |
| TPE_0114 | possible HflC protein | ***hflC*** | 331 | General metabolism |
| TPE_0115 | phosphomethypyrimidine kinase | ***thiD*** | 269 | General metabolism |
| TPE_0122 | phosphoenolpyruvate carboxykinase (GTP) | ***pckG*** | 618 | General metabolism |
| TPE_0153 | possible phosphatidate phosphatase |  | 163 | General metabolism |
| TPE_0156 | possible 4-hydroxybenzoyl-CoA thioesterase |  | 134 | General metabolism |
| TPE_0157 | probable phosphate acyltransferase | ***plsC*** | 300 | General metabolism |
| TPE_0158 | HAD-superfamily hydrolase |  | 228 | General metabolism |
| TPE_0168 | phosphoglycerate mutase family protein |  | 251 | General metabolism |
| TPE_0170 | nucleoside phosphorylase |  | 269 | General metabolism |
| TPE_0186 | coproporphyrinogen dehydrogenase | ***hemN*** | 405 | General metabolism |
| TPE_0223 | pyridoxal phosphate-dependent aspartate aminotransferase |  | 479 | General metabolism |
| TPE_0228 | BioY protein | ***bioY*** | 192 | General metabolism |
| TPE_0256 | CDP-diacylglycerol--glycerol-3-phosphate 3-phosphatidyltransferase | ***pgsA*** | 216 | General metabolism |
| TPE_0257 | glycerophosphodiester phosphodiesterase | ***glpQ*** | 356 | General metabolism |
| TPE_0264 | deoxyribose-phosphate aldolase | ***deoC*** | 218 | General metabolism |
| TPE_0274 | dCMP deaminase | ***dctD*** | 166 | General metabolism |
| TPE_0275 | WecB/TagA/CpsF family glycosyl transferase | ***wecG*** | 249 | General metabolism |
| TPE_0276 | possible RNA methyltransferase |  | 279 | General metabolism |
| TPE_0283 | pantetheine-phosphate adenylyltransferase | ***coaD*** | 159 | General metabolism |
| TPE_0290 | HAD superfamily hydrolase |  | 277 | General metabolism |
| TPE_0291 | probable FMN-dependent family dehydrogenase |  | 293 | General metabolism |
| TPE_0294 | ribose-phosphate diphosphokinase | ***prs*** | 421 | General metabolism |
| TPE_0295 | FGGY family carhohydrate kinase |  | 414 | General metabolism |
| TPE_0296 | probable dephospho-CoA kinase | ***coaE*** | 211 | General metabolism |
| TPE_0305 | CTP synthase | ***pyrG*** | 577 | General metabolism |
| TPE_0329 | glycine hydroxymethyltransferase | ***glyA*** | 513 | General metabolism |
| TPE_0330 | AAA family ATPase |  | 598 | General metabolism |
| TPE_0331 | phosphogluconate dehydrogenase (decarboxylating) | ***gnd*** | 488 | General metabolism |
| TPE_0340 | bifunctional dihydrofolate synthase/tetrahydrofolate synthase | ***folC*** | 490 | General metabolism |
| TPE_0342 | cytidylate kinase | ***cmk*** | 186 | General metabolism |
| TPE_0354 | dTMP kinase | ***tmk*** | 208 | General metabolism |
| TPE_0357 | biotin--[acetyl-CoA-carboxylase] ligase | ***birA*** | 219 | General metabolism |
| TPE_0361 | acyltransferase family protein |  | 267 | General metabolism |
| TPE_0407 | HAD family hydrolase |  | 226 | General metabolism |
| TPE_0413 | phosphohexomutase |  | 632 | General metabolism |
| TPE_0418 | galactokinase | ***galK*** | 397 | General metabolism |
| TPE_0436 | DHH superfamily phosphoesterase |  | 360 | General metabolism |
| TPE_0438 | HAM1 protein |  | 269 | General metabolism |
| TPE_0448 | uracil phosphoribosyltransferase | ***upp*** | 360 | General metabolism |
| TPE_0476 | acetate kinase | ***ackA*** | 448 | General metabolism |
| TPE_0477 | 6-phosphogluconolactonase |  | 234 | General metabolism |
| TPE_0478 | glucose-6-phosphate 1-dehydrogenase | ***zwf*** | 515 | General metabolism |
| TPE_0485 | probable adenylate cyclase |  | 614 | General metabolism |
| TPE_0505 | hexokinase |  | 444 | General metabolism |
| TPE_0509 | peroxiredoxin | ***ahpC*** | 188 | General metabolism |
| TPE_0518 | thiamine pyrophosphokinase |  | 230 | General metabolism |
| TPE_0537 | triose-phosphate isomerase | ***tpiA*** | 249 | General metabolism |
| TPE_0538 | phosphoglycerate kinase | ***pgk*** | 419 | General metabolism |
| TPE_0546 | S1B subfamily peptidase |  | 571 | General metabolism |
| TPE_0551 | membrane phosphatase |  | 362 | General metabolism |
| TPE_0554 | phosphoglycolate phosphatase | ***gph*** | 247 | General metabolism |
| TPE_0560 | transketolase | ***tktA*** | 661 | General metabolism |
| TPE_0568 | bifunctional 4-hydroxy-2-oxoglutarate aldolase/2-dehydro-3-deoxy-phosphogluconate aldolase | ***eda*** | 210 | General metabolism |
| TPE_0591 | bifunctional Hpr kinase/phosphatase | ***hprK*** | 329 | General metabolism |
| TPE_0595 | adenylate kinase | ***adk*** | 211 | General metabolism |
| TPE_0601 | 1-deoxy-D-xylulose-5-phosphate reductoisomerase | ***dxr*** | 376 | General metabolism |
| TPE_0602 | phosphatidate cytidylyltransferase | ***cdsA*** | 287 | General metabolism |
| TPE_0603 | di-trans,poly-cis-decaprenylcistransferase | ***uppS*** | 228 | General metabolism |
| TPE_0615 | SUF system FeS assembly protein |  | 147 | General metabolism |
| TPE_0616 | ribose 5-phosphate isomerase A | ***rpiA*** | 262 | General metabolism |
| TPE_0628 | nicotinate phosphoribosyltransferase | ***pncB*** | 493 | General metabolism |
| TPE_0662 | fructose-bisphosphate aldolase | ***fbaB*** | 332 | General metabolism |
| TPE_0683 | octaprenyl-diphosphate synthase |  | 377 | General metabolism |
| TPE_0694 | 5-formyltetrahydrofolate cyclo-ligase |  | 225 | General metabolism |
| TPE_0702 (TP0702, TP0703) | M23B subfamily peptidase |  | 410 | General metabolism |
| TPE_0706 | M23B subfamily peptidase |  | 325 | General metabolism |
| TPE_0730 | CDP-diacylglycerol--glycerol-3-phosphate 3-phosphatidyltransferase | ***pgsA*** | 365 | General metabolism |
| TPE_0731 | ADP-ribose diphosphatase | ***nudE*** | 206 | General metabolism |
| TPE_0732 | bifunctional methylenetetrahydrofolate dehydrogenase (NADP(+))/methenyltetrahydrofolate cyclohydrolase | ***folD*** | 315 | General metabolism |
| TPE_0734 | purine-nucleoside phosphorylase | ***deoD*** | 233 | General metabolism |
| TPE_0736 | ferredoxin--NADP(+) reductase, subunit alpha |  | 280 | General metabolism |
| TPE_0741 | probable nicotinate-nucleotide adenylyltransferase | ***nadD*** | 204 | General metabolism |
| TPE_0773 | S1 family peptidase Do | ***htrA*** | 303 | General metabolism |
| TPE_0778 | probable nucleoside-triphosphate diphosphatase | ***mazG*** | 396 | General metabolism |
| TPE_0794 | methionine adenosyltransferase | ***metK*** | 362 | General metabolism |
| TPE_0796 | conserved hypothetical protein |  | 263 | General metabolism |
| TPE_0797 | pyrroline-5-carboxylate reductase | ***proC*** | 78 | General metabolism |
| TPE_0808 | acyl carrier protein | ***acpP*** | 327 | General metabolism |
| TPE_0815 | possible GNAT family acetyltransferase |  | 327 | General metabolism |
| TPE_0817 | phosphopyruvate hydratase | ***eno*** | 432 | General metabolism |
| TPE_0819 | ribonuclease Z | ***rnz*** | 308 | General metabolism |
| TPE_0823 | probable superoxide reductase | ***sorA*** | 128 | General metabolism |
| TPE_0824 | 1-deoxy-D-xylulose-5-phosphate synthase | ***dxs*** | 630 | General metabolism |
| TPE_0828 | holo-[acyl-carrier-protein] synthase | ***acpS*** | 125 | General metabolism |
| TPE_0844 | glyceraldehyde-3-phosphate dehydrogenase | ***gap*** | 350 | General metabolism |
| TPE_0862 | peptidylprolyl isomerase | ***fklB*** | 264 | General metabolism |
| TPE_0863 | bifunctional cysteine sulfinate desulfinase/cysteine desulfurase | ***nifS*** | 427 | General metabolism |
| TPE_0885 | dUTP diphosphatase | ***dut*** | 146 | General metabolism |
| TPE_0886 | polyribonucleotide nucleotidyltransferase | ***pnp*** | 702 | General metabolism |
| TPE_0888 | bifunctional riboflavin kinase/FAD synthetase | ***ribF*** | 275 | General metabolism |
| TPE_0902 | probable esterase/lipase |  | 293 | General metabolism |
| TPE_0906 | possible RNA-binding protein |  | 80 | General metabolism |
| TPE_0919 | thioredoxin group 1 family protein |  | 105 | General metabolism |
| TPE_0921 | NADH dehydrogenase | ***ndh*** | 445 | General metabolism |
| TPE_0925 | nitrogenase (flavodoxin) |  | 146 | General metabolism |
| TPE_0937 | probable phosphoesterase |  | 211 | General metabolism |
| TPE_0939 | pyruvate synthase |  | 1184 | General metabolism |
| TPE_0945 | ribulose-phosphate 3-epimerase | ***rpe*** | 218 | General metabolism |
| TPE_0952 | possible lipase/esterase |  | 345 | General metabolism |
| TPE_0975 | possible tetrapyrrole methylase |  | 274 | General metabolism |
| TPE_0979 | magnesium (Mg2+)-dependent deoxyribonuclease | ***tatD*** | 257 | General metabolism |
| TPE_0982 | rhomboid family protein |  | 208 | General metabolism |
| TPE_0991 | rubredoxin |  | 52 | General metabolism |
| TPE_0997 | signal peptide peptidase A | ***sppA*** | 602 | General metabolism |
| TPE_1007 | thymidylate synthase (FAD) | ***thyX*** | 305 | General metabolism |
| TPE_1008 | ribonucleotide-diphosphate reductase subunit alpha | ***nrdA*** | 845 | General metabolism |
| TPE_1009 | glycerol-3-phosphate dehydrogenase (NAD(P)(+)) | ***gpsA*** | 356 | General metabolism |
| TPE_1010 | nucleoside-diphosphate kinase | ***ndk*** | 149 | General metabolism |
| TPE_1026 | possible oligoendopeptidase F |  | 589 | General metabolism |
| TPE_1027 | uridine phosphorylase | ***udp*** | 258 | General metabolism |
| TPE_0082 | sigma-54 dependent transcriptional regulator | ***fhlA*** | 528 | Regulation |
| TPE_0089 | cAMP-dependent protein kinase |  | 411 | Regulation |
| TPE_0093 | possible transcriptional regulator (anti-sigma factor) |  | 215 | Regulation |
| TPE_0096 | probable DnaK suppressor protein | ***dksA*** | 120 | Regulation |
| TPE_0167 | iron (Fe2+)/zinc (Zn2+)/manganese (Mn2+)-dependent transcriptional regulator | ***troR*** | 153 | Regulation |
| TPE_0218 | probable sigma factor regulatory protein | ***rsbU*** | 489 | Regulation |
| TPE_0219 | probable sigma factor regulatory protein | ***rsbU*** | 715 | Regulation |
| TPE_0220 | anti-sigma factor antagonist |  | 108 | Regulation |
| TPE_0233 | anti-sigma factor antagonist |  | 181 | Regulation |
| TPE_0236 | transcription antitermination protein NusG | ***nusG*** | 185 | Regulation |
| TPE_0271 | chromosome partitioning protein | ***parB*** | 324 | Regulation |
| TPE_0272 | chromosome partitioning protein | ***parA*** | 253 | Regulation |
| TPE_0363 | sensor histidine kinase | ***cheA*** | 812 | Regulation |
| TPE_0365 | probable chemotaxis protein CheX | ***cheX*** | 154 | Regulation |
| TPE_0366 | response regulator | ***cheY*** | 144 | Regulation |
| TPE_0431 | possible Baf family transcriptional regulator |  | 273 | Regulation |
| TPE_0439 | chemotaxis protein CheW | ***cheW*** | 170 | Regulation |
| TPE_0461 | probable transcriptional regulator |  | 119 | Regulation |
| TPE_0511 | CarD family transcriptional regulator |  | 243 | Regulation |
| TPE_0519 | sigma-54 dependent response regulator |  | 458 | Regulation |
| TPE_0520 | sensor histidine kinase |  | 395.6667 | Regulation |
| TPE_0540 | anti sigma factor antagonist |  | 121 | Regulation |
| TPE_0657 | carbon storage regulator | ***csrA*** | 73 | Regulation |
| TPE_0689 | GTP-binding protein | ***engA*** | 460 | Regulation |
| TPE_0720 | bifunctional chemotaxis protein CheC/flagellar motor switch protein FliY | ***fliY*** | 348 | Regulation |
| TPE_0742 | GTP-binding protein | ***obgE*** | 376 | Regulation |
| TPE_0764 | conserved hypothetical protein |  | 391 | Regulation |
| TPE_0995 | possible CAP family of transcription factors |  | 444 | Regulation |
| TPE_1015 | transcription antitermination protein NusB | ***nusB*** | 141 | Regulation |
| TPE_1023 | recombination regulator RecX | ***recX*** | 222 | Regulation |
| TPE_0092 | DNA-directed RNA polymerase sigma subunit RpoE | ***rpoE*** | 180 | Transcription |
| TPE_0254 | transcription termination factor Rho | ***rho*** | 519 | Transcription |
| TPE_0493 | DNA-directed RNA polymerase sigma subunit RpoD | ***rpoD*** | 611 | Transcription |
| TPE_0701 | probable DNA-directed RNA polymerase subunit omega | ***rpoZ*** | 67 | Transcription |
| TPE_0809 | ribonuclease III | ***rnc*** | 254 | Transcription |
| TPE_0892 | transcription elongation factor | ***nusA*** | 485 | Transcription |
| TPE_1012 | DNA-directed RNA polymerase sigma subunit RpoD family | ***sigA*** | 311 | Transcription |
| TPE_0015 | phenylalanine--tRNA ligase, beta subunit | ***pheT*** | 604 | Translation |
| TPE_0044 | glucose-inhibited division protein A | ***gidA*** | 630 | Translation |
| TPE_0051 | peptide chain release factor RF1 | ***prfA*** | 351 | Translation |
| TPE_0052 | HemK family methyltransferase | ***hemK*** | 338 | Translation |
| TPE_0060 | ribosomal protein L9 | ***rplI*** | 156 | Translation |
| TPE_0061 | ribosomal protein S18 | ***rpsR*** | 99 | Translation |
| TPE_0063 | ribosomal protein S6 | ***rpsF*** | 93 | Translation |
| TPE_0091 | cysteine--tRNA ligase | ***cysS*** | 520 | Translation |
| TPE_0097 | initiation factor IF1 | ***infA*** | 72 | Translation |
| TPE_0109 | probable rRNA (adenosine-2'-O-)-methyltransferase | ***spoU*** | 292 | Translation |
| TPE_0124 | Obg family GTP-binding protein | ***ychF*** | 368 | Translation |
| TPE_0154 | pseudouridylate synthase | ***rluA*** | 404 | Translation |
| TPE_0160 | proline--tRNA ligase | ***proS*** | 619 | Translation |
| TPE_0182 | Sua5/YciO/YrdC/YwlC family protein | ***sua5*** | 212 | Translation |
| TPE_0184 | SsrA-binding protein | ***smpB*** | 154 | Translation |
| TPE_0187 | elongation factor EF1A | ***tufA*** | 395 | Translation |
| TPE_0188 | ribosomal protein S10 | ***rpsJ*** | 102 | Translation |
| TPE_0189 | ribosomal protein L3 | ***rplC*** | 208 | Translation |
| TPE_0191 | ribosomal protein L23 | ***rplW*** | 94 | Translation |
| TPE_0192 | ribosomal protein L2 | ***rplB*** | 273 | Translation |
| TPE_0193 | ribosomal protein S19 | ***rpsS*** | 95 | Translation |
| TPE_0195 | ribosomal protein S3 | ***rpsC*** | 247 | Translation |
| TPE_0196 | ribosomal protein L16 | ***rplP*** | 139 | Translation |
| TPE_0199 | ribosomal protein L14 | ***rplN*** | 122 | Translation |
| TPE_0200 | ribosomal protein L24 | ***rplX*** | 103 | Translation |
| TPE_0201 | ribosomal protein L5 | ***rplE*** | 185 | Translation |
| TPE_0202 | ribosomal protein S14 | ***rpsN*** | 61 | Translation |
| TPE_0203 | ribosomal protein S8 | ***rpsH*** | 132 | Translation |
| TPE_0204 | probable ribosomal protein L6 | ***rplF*** | 179 | Translation |
| TPE_0205 | ribosomal protein L18 | ***rplR*** | 120 | Translation |
| TPE_0206 | ribosomal protein S5 | ***rpsE*** | 172 | Translation |
| TPE_0206a | ribosomal protein L30 | ***rpmD*** | 61 | Translation |
| TPE_0207 | ribosomal protein L15 | ***rplO*** | 153 | Translation |
| TPE_0209 | ribosomal protein L36 | ***rpmJ*** | 37 | Translation |
| TPE_0210 | ribosomal protein S13 | ***rpsM*** | 121 | Translation |
| TPE_0211 | ribosomal protein S11 | ***rpsK*** | 126 | Translation |
| TPE_0213 | ribosomal protein L17 | ***rplQ*** | 164 | Translation |
| TPE_0231 | pseudouridylate synthase |  | 216 | Translation |
| TPE_0234 | ribosomal protein L33 | ***rpmG*** | 56 | Translation |
| TPE_0237 | ribosomal protein L11 | ***rplK*** | 146 | Translation |
| TPE_0238 | ribosomal protein L1 | ***rplA*** | 226 | Translation |
| TPE_0239 | ribosomal protein L10 | ***rplJ*** | 180 | Translation |
| TPE_0240 | ribosomal protein L7/L12 | ***rplL*** | 129 | Translation |
| TPE_0243 | ribosomal protein S12 | ***rpsL*** | 124 | Translation |
| TPE_0244 | ribosomal protein S7 | ***rpsG*** | 156 | Translation |
| TPE_0255 | ribosomal protein L31 | ***rpmE*** | 67 | Translation |
| TPE_0270 | polynucleotide adenylyltransferase | ***pcnB*** | 508 | Translation |
| TPE_0279 | bifunctional cytidylate kinase/ribosomal protein S1 |  | 863 | Translation |
| TPE_0306 | ribosomal protein S4 | ***rpsD*** | 204 | Translation |
| TPE_0337 | rRNA dimethyladenosine transferase | ***ksgA*** | 285 | Translation |
| TPE_0339 | pseudouridylate synthase | ***rluA*** | 331 | Translation |
| TPE_0362 | ribosomal protein L28 | ***rpmB*** | 78 | Translation |
| TPE_0372 | ribosomal 5S rRNA E-loop binding protein Ctc/L25/TL5 |  | 196 | Translation |
| TPE_0450 | elongation factor EF2 | ***fusA*** | 683 | Translation |
| TPE_0459 | RsuA family pseudouridine synthase | ***rluB*** | 265 | Translation |
| TPE_0525 | elongation factor P | ***efp*** | 187 | Translation |
| TPE_0576 | peptide chain release factor RF2 | ***prfB*** | 368 | Translation |
| TPE_0596 | tRNA polynucleotide adenylyltransferase | ***pcnB*** | 370 | Translation |
| TPE_0604 | ribosome recycling factor | ***frr*** | 183 | Translation |
| TPE_0605 | elongation factor EF1B | ***tsf*** | 290 | Translation |
| TPE_0606 | ribosomal protein S2 | ***rpsB*** | 291 | Translation |
| TPE_0632 | tryptophan--tRNA ligase | ***trpS*** | 337 | Translation |
| TPE_0637 | tRNA isopentenyltransferase | ***miaA*** | 316 | Translation |
| TPE_0641 | histidine--tRNA ligase | ***hisS*** | 442 | Translation |
| TPE_0644 | lysine--tRNA ligase | ***lysS*** | 528 | Translation |
| TPE_0647 | serine--tRNA ligase | ***serS*** | 426 | Translation |
| TPE_0672 | glycine--tRNA ligase | ***glyS*** | 491 | Translation |
| TPE_0673 | glutamate--tRNA ligase | ***gltX*** | 537 | Translation |
| TPE_0682 | 23S rRNA methyltransferase | ***rrmJ*** | 200 | Translation |
| TPE_0743 | ribosomal protein L27 | ***rpmA*** | 87 | Translation |
| TPE_0745 | ribosomal protein L21 | ***rplU*** | 111 | Translation |
| TPE_0754 | 2-methylthioadenine synthetase | ***miaB*** | 456 | Translation |
| TPE_0756 | methionyl-tRNA formyltransferase | ***fmt*** | 319 | Translation |
| TPE_0757 | peptide deformylase | ***def*** | 162 | Translation |
| TPE_0758 | ribosomal protein S21 | ***rpsU*** | 69 | Translation |
| TPE_0767 | elongation factor EF2 | ***fusA*** | 695 | Translation |
| TPE_0798 | methionine--tRNA ligase | ***metG*** | 811 | Translation |
| TPE_0807 | ribosomal protein L32 | ***rpmF*** | 62 | Translation |
| TPE_0834 | tyrosine--tRNA ligase | ***tyrS*** | 409 | Translation |
| TPE_0837 | threonine--tRNA ligase | ***thrS*** | 592 | Translation |
| TPE_0842 | methionyl aminopeptidase | ***map*** | 255 | Translation |
| TPE_0848 | ribosomal protein L20 | ***rplT*** | 122 | Translation |
| TPE_0850 | initiation factor IF3 | ***infC*** | 173 | Translation |
| TPE_0887 | ribosomal protein S15 | ***rpsO*** | 89 | Translation |
| TPE_0890 | ribosome-binding factor A | ***rbfA*** | 126 | Translation |
| TPE_0891 | initiation factor IF2 | ***infB*** | 898 | Translation |
| TPE_0905 | ribosomal protein S16 | ***rpsP*** | 123 | Translation |
| TPE_0907 | probable 16S rRNA processing protein RimM | ***rimM*** | 178 | Translation |
| TPE_0908 | tRNA (guanine-N(1)-)-methyltransferase | ***trmD*** | 250 | Translation |
| TPE_0909 | ribosomal protein L19 | ***rplS*** | 123 | Translation |
| TPE_0951 | ribosomal protein L34 | ***rpmH*** | 51 | Translation |
| TPE_0973 | phenylalanine--tRNA ligase alpha subunit | ***pheS*** | 549 | Translation |
| TPE_0980 | tRNA-dihydrouridine synthase | ***dus*** | 340 | Translation |
| TPE_0985 | aspartate--tRNA ligase | ***aspS*** | 589 | Translation |
| TPE_1011 | aminoacyl-tRNA hydrolase | ***pth*** | 202 | Translation |
| TPE_1017 | alanine--tRNA ligase | ***alaS*** | 605 | Translation |
| TPE_1019 | aspartyl/glutamyl-tRNA(Asn/Gln) amidotransferase subunit C | ***gatC*** | 147 | Translation |
| TPE_1021 | aspartyl/glutamyl-tRNA(Asn/Gln) amidotransferase subunit B | ***gatB*** | 509 | Translation |
| TPE_1025 | ribosomal protein L13 | ***rplM*** | 142 | Translation |
| TPE_1040 | lysine--tRNA ligase | ***lysS*** | 351 | Translation |
| TPE_0023 | NSS family probable amino acid:sodium (Na+) symporter |  | 443 | Transport |
| TPE_0024 | Trk family potassium (K+) transporter, NAD+ binding protein |  | 236 | Transport |
| TPE_0034 | zinc (Zn2+) ABC superfamily ATP binding cassette transporter, binding protein | ***znuA*** | 376 | Transport |
| TPE_0035 | zinc (Zn2+) ABC superfamily ATP binding cassette transporter, ABC protein | ***znuC*** | 238 | Transport |
| TPE_0036 | zinc (Zn2+) ABC superfamily ATP binding cassette transporter, membrane protein | ***znuB*** | 266 | Transport |
| TPE_0055 | oxaloacetate decarboxylase subunit gamma | ***oadG*** | 78 | Transport |
| TPE_0057 | oxaloacetate decarboxylase subunit beta | ***oadB*** | 469 | Transport |
| TPE_0074 | sugar ABC superfamily ATP binding cassette transporter, binding protein | ***ugpB*** | 469 | Transport |
| TPE_0075 | sugar ABC superfamily ATP binding cassette transporter, membrane protein | ***ugpA*** | 296 | Transport |
| TPE_0076 | sugar ABC superfamily ATP binding cassette transporter, membrane protein | ***ugpE*** | 276 | Transport |
| TPE_0085 | PTS family fructose (fru) porter component IIA |  | 148 | Transport |
| TPE_0106 | BCCT family betaine/carnitine/choline transporter | ***betT*** | 510 | Transport |
| TPE_0119 | methionine ABC superfamily ATP binding cassette transporter, membrane protein | ***metI*** | 219 | Transport |
| TPE_0120 | methionine ABC superfamily ATP binding cassette transporter, ABC protein | ***metN*** | 269 | Transport |
| TPE_0139 | Trk family potassium (K+) transporter, NAD+ binding protein |  | 230 | Transport |
| TPE_0142 | possible sugar ABC superfamily ATP binding cassette transporter, ABC protein |  | 220 | Transport |
| TPE_0144 | thiamine ABC superfamily ATP binding cassette transporter, binding protein | ***tbpA*** | 335 | Transport |
| TPE_0163 | iron (Fe2+)/zinc (Zn2+)/manganese (Mn2+) ABC superfamily ATP binding cassette transporter, binding protein | ***troA*** | 308 | Transport |
| TPE_0165 | iron (Fe2+)/zinc (Zn2+)/manganese (Mn2+) ABC superfamily ATP binding cassette transporter, membrane protein | ***troC*** | 298 | Transport |
| TPE_0166 | iron (Fe2+)/zinc (Zn2+)/manganese (Mn2+) ABC superfamily ATP binding cassette transporter, membrane protein | ***troD*** | 367 | Transport |
| TPE_0235 | Sec family Type I general secretory pathway protein, subunit SecE | ***secE*** | 59 | Transport |
| TPE_0292 | OOP family OmpA-OmpF porin |  | 417 | Transport |
| TPE_0298 | ABC superfamily ATP binding cassette transporter, membrane protein |  | 348 | Transport |
| TPE_0301 | sugar ABC superfamily ATP binding cassette transporter, membrane protein |  | 377 | Transport |
| TPE_0302 | sugar ABC superfamily ATP binding cassette transporter, membrane protein |  | 313 | Transport |
| TPE_0308 | probable polar amino acid ABC superfamily ATP binding cassette transporter, binding protein |  | 321 | Transport |
| TPE_0309 | probable polar amino acid ABC superfamily ATP binding cassette transporter, binding protein |  | 272 | Transport |
| TPE_0322 | sugar ABC superfamily ATP binding cassette transporter, membrane protein |  | 400 | Transport |
| TPE_0323 | sugar ABC superfamily ATP binding cassette transporter, membrane protein |  | 316 | Transport |
| TPE_0410 | RND superfamily resistance-nodulation-cell division protein:proton (H+) antiporter | ***secD*** | 583 | Transport |
| TPE_0411 | RND superfamily resistance-nodulation-cell division protein:proton (H+) antiporter | ***secF*** | 420 | Transport |
| TPE_0414 | AGCS family alanine or glycine:sodium (Na+) or proton (H+) symporter | ***alsT*** | 451 | Transport |
| TPE_0424 | H(+) transporting two-sector ATPase, V(1) subunit E | ***ntpE*** | 204 | Transport |
| TPE_0426 | two-sector ATPase, V(1) subunit A | ***ntpA*** | 589 | Transport |
| TPE_0427 | two-sector ATPase, V(1) subunit B | ***ntpB*** | 430 | Transport |
| TPE_0428 | two-sector ATPase, V(1) subunit D | ***ntpD*** | 206 | Transport |
| TPE_0430 | two-sector ATPase, V(0) subunit K | ***ntpK*** | 140 | Transport |
| TPE_0513 | Trk family potassium (K+) transporter, membrane protein | ***trkA*** | 466 | Transport |
| TPE_0516 | integral membrane protein MviN | ***mviN*** | 526 | Transport |
| TPE_0527 | two-sector ATPase, V(1) subunit D | ***ntpD*** | 209 | Transport |
| TPE_0528 | two-sector ATPase, V(1) subunit B | ***ntpB*** | 480 | Transport |
| TPE_0529 | two-sector ATPase, V(1) subunit A | ***ntpA*** | 605 | Transport |
| TPE_0531 | two-sector ATPase, V(1) subunit F |  | 105 | Transport |
| TPE_0532 | not annotated in TPE, V-type ATPase, subunit K, authentic frameshift in Nichols | ***atpK-2*** | 155.67 | Transport |
| TPE_0533 | two-sector ATPase, V(0) subunit I | ***ntpI*** | 561 | Transport |
| TPE_0545 | sugar ABC superfamily ATP binding cassette transporter, binding protein |  | 354 | Transport |
| TPE_0555 | DAACS family dicarboxylate/amino acid:sodium (Na+) or proton (H+) symporter | ***gltP*** | 393 | Transport |
| TPE_0558 | probable NiCoT family nickel (Ni2+)-cobalt (Co2+) transporter | ***nicO*** | 302 | Transport |
| TPE_0578 | Sec family Type I general secretory pathway protein | ***ftsY*** | 295 | Transport |
| TPE_0580 | probable lipoprotein ABC superfamily ATP binding cassette transporter, membrane protein | ***lolE*** | 429 | Transport |
| TPE_0581 | lipoprotein/macrolide ABC superfamily ATP binding cassette transporter, ABC protein |  | 226 | Transport |
| TPE_0582 | lipoprotein ABC superfamily ATP binding cassette transporter, membrane protein | ***lolE*** | 495 | Transport |
| TPE_0589 | HPr family phosphotransferase system protein | ***hpr*** | 88 | Transport |
| TPE_0611 | ABC superfamily ATP binding cassette transporter, ABC protein | ***sufC*** | 259 | Transport |
| TPE_0612 | ABC superfamily ATP binding cassette transporter, membrane protein | ***sufB*** | 479 | Transport |
| TPE_0613 | ABC superfamily ATP binding cassette transporter, membrane protein | ***sufD*** | 382 | Transport |
| TPE_0624 | OOP family OmpA-OmpF porin |  | 476 | Transport |
| TPE_0652 | spermidine/putrescine ABC superfamily ATP binding cassette transporter, ABC protein | ***potA*** | 375 | Transport |
| TPE_0654 | spermidine/putrescine ABC superfamily ATP binding cassette transporter, membrane protein | ***potC*** | 272 | Transport |
| TPE_0655 | spermidine/putrescine ABC superfamily ATP binding cassette transporter, binding protein | ***potD*** | 348 | Transport |
| TPE_0686 | galactose ABC superfamily ATP binding cassette transporter, membrane protein | ***mglC*** | 531 | Transport |
| TPE_0724 | Mot family proton (H+) or sodium (Na+) transporter MotB | ***motB*** | 240 | Transport |
| TPE_0725 | Mot family proton (H+) or sodium (Na+) transporter MotA | ***motA*** | 259 | Transport |
| TPE_0737 | sugar ABC superfamily ATP binding cassette transporter, binding protein | ***msmE*** | 436 | Transport |
| TPE_0755 | PTS family fructose/mannitol (fru) porter component IIA | ***ptsN*** | 218 | Transport |
| TPE_0774 | probable MgtC family magnesium (Mg2+) transporter-C | ***mgtC*** | 224 | Transport |
| TPE_0786 | ABC superfamily ATP binding cassette transporter, ABC protein |  | 237 | Transport |
| TPE_0790 | probable RND superfamily resistance-nodulation-cell division antibiotic:proton (H+) antiporter |  | 888 | Transport |
| TPE_0804 | sugar ABC superfamily ATP binding cassette transporter, ABC protein |  | 387 | Transport |
| TPE_0821 | probable metal ion ABC superfamily ATP binding cassette transporter, binding protein | ***tpn32*** | 268 | Transport |
| TPE_0822 | MscS family small conductance mechanosenstive ion channel |  | 301 | Transport |
| TPE_0880 | ABC superfamily ATP binding cassette transporter, membrane protein |  | 241 | Transport |
| TPE_0881 | ABC superfamily ATP binding cassette transporter, ABC protein | ***natA*** | 268 | Transport |
| TPE_0901 | MATE family multi antimicrobial extrusion protein OR | ***norM*** | 470 | Transport |
| TPE_0957 | TRAP-T family tripartite ATP-independent periplasmic transporter, binding protein |  | 342 | Transport |
| TPE_0962 | possible macrolide ABC superfamily ATP binding cassette transporter, ABC/membrane protein |  | 410 | Transport |
| TPE_0963 | possible macrolide ABC superfamily ATP binding cassette transporter, ABC/membrane protein |  | 409 | Transport |
| TPE_0964 | possible macrolide ABC superfamily ATP binding cassette transporter, ABC protein |  | 235 | Transport |
| TPE_0965 | membrane-fusion protein | ***macA*** | 320 | Transport |
| TPE_0986 | DMT superfamily drug/metabolite transporter |  | 294 | Transport |
| TPE_0011 | Tpr protein B |  | 660 | Potential virulence factor |
| TPE_0171 | lipoprotein, 15 kDa (tpp15) | ***tpp15*** |  | Virulence |
| TPE_0263 | conserved hypothetical protein |  |  | Virulence |
| TPE_0399 | IIISP family Type III (virulence-related) secretory pathway protein | ***fliF*** |  | Virulence |
| TPE_0401 | IIISP family Type III (virulence-related) secretory pathway protein | ***fliH*** |  | Virulence |
| TPE_0402 | IIISP family Type III (virulence-related) secretory pathway protein | ***fliI*** |  | Virulence |
| TPE_0714 | IIISP family Type III (virulence-related) secretory pathway protein | ***flhA*** | 0.167 | Virulence |
| TPE_0716 | IIISP family Type III (virulence-related) secretory pathway protein | ***fliR*** | 0.265 | Virulence |
| TPE_0717 | IIISP family Type III (virulence-related) secretory pathway protein | ***fliQ*** | 0.359 | Virulence |
| TPE_0718 | IIISP family Type III (virulence-related) secretory pathway protein | ***fliP*** | 0.849 | Virulence |
| TPE_0751 | laminin-binding protein |  |  | Virulence |
| TPE_0971 | tp34 lipoprotein | ***tpd*** |  | Virulence |
| TPE_0004 | treponemal conserved hypothetical protein |  | 145 | Unknown |
| TPE_0006 (TP0006, TP0007, TP0008) | conserved hypothetical protein |  | 415 | Unknown |
| TPE_0012 | hypothetical protein |  | 58 | Unknown |
| TPE_0013 (TP0013, TP0014) | conserved hypothetical protein |  | 410 | Unknown |
| TPE_0017 | conserved hypothetical protein |  | 315 | Unknown |
| TPE_0020 | treponemal conserved hypothetical membrane protein |  | 707 | Unknown |
| TPE_0021 | hypothetical protein |  | 88 | Unknown |
| TPE_0022 | hypothetical protein |  | 681 | Unknown |
| TPE_0032 | conserved hypothetical protein |  | 266 | Unknown |
| TPE_0033 | conserved hypothetical membrane protein |  | 222 | Unknown |
| TPE_0039 | hypothetical protein |  | 59 | Unknown |
| TPE_0042 | treponemal conserved hypothetical protein |  | 310 | Unknown |
| TPE_0046 | conserved hypothetical protein |  | 233 | Unknown |
| TPE_0047 | conserved hypothetical protein |  | 173 | Unknown |
| TPE_0048 | conserved hypothetical protein |  | 143 | Unknown |
| TPE_0059 | hypothetical protein |  | 75 | Unknown |
| TPE_0064 | treponemal conserved hypothetical protein |  | 196 | Unknown |
| TPE_0070 | treponemal conserved hypothetical membrane protein |  | 128 | Unknown |
| TPE_0073 | conserved hypothetical protein |  | 513 | Unknown |
| TPE_0084 | hypothetical protein |  | 77 | Unknown |
| TPE_0087 | conserved hypothetical protein |  | 187 | Unknown |
| TPE_0088 | conserved hypothetical protein |  | 204 | Unknown |
| TPE_0095 | conserved hypothetical protein |  | 648 | Unknown |
| TPE_0118 | treponemal conserved hypothetical protein |  | 421 | Unknown |
| TPE_0126 | treponemal conserved hypothetical protein |  | 223 (458 u SD) | Unknown |
| TPE_0127a | hypothetical protein |  | 126 | Unknown |
| TPE_0127b | hypothetical protein |  | 137 | Unknown |
| TPE_0128 | hypothetical protein |  | 115 | Unknown |
| TPE_0138 | treponemal conserved hypothetical membrane protein |  | 245 | Unknown |
| TPE_0150 | treponemal conserved hypothetical protein |  | 161 | Unknown |
| TPE_0159 | treponemal conserved hypothetical protein |  | 342 | Unknown |
| TPE_0172 (TP0172, TP0173) | conserved hypothetical membrane protein |  | 459 | Unknown |
| TPE_0174 (TP0174, TP0175, TP0176) | conserved hypothetical protein |  | 506 | Unknown |
| TPE_0177 | conserved hypothetical protein |  | 470 | Unknown |
| TPE_0178 | treponemal conserved hypothetical protein |  | 308 | Unknown |
| TPE_0179 | treponemal conserved hypothetical protein |  | 649 | Unknown |
| TPE_0183 | treponemal conserved hypothetical protein |  | 281 | Unknown |
| TPE_0214 | treponemal conserved hypothetical protein |  | 65 | Unknown |
| TPE_0222 | hypothetical protein |  | 148 | Unknown |
| TPE_0245 | conserved hypothetical protein |  | 1151 | Unknown |
| TPE_0246 | conserved hypothetical protein |  | 650 | Unknown |
| TPE_0250 | hypothetical membrane protein |  | 75 | Unknown |
| TPE_0253 | conserved hypothetical protein |  | 169 | Unknown |
| TPE_0258 | conserved hypothetical protein |  | 236 | Unknown |
| TPE_0267 | conserved hypothetical protein |  | 121 | Unknown |
| TPE_0273 | hypothetical membrane protein |  | 263 | Unknown |
| TPE_0282 | treponemal conserved hypothetical protein |  | 239 | Unknown |
| TPE_0284 (TP0284, TP0285) | conserved hypothetical protein |  | 559 | Unknown |
| TPE_0286 (TP0286, TP0287) | conserved hypothetical protein |  | 318 | Unknown |
| TPE_0293 | hypothetical protein |  | 58 | Unknown |
| TPE_0297 | conserved hypothetical protein |  | 270 | Unknown |
| TPE_0307 | conserved hypothetical protein |  | 349 | Unknown |
| TPE_0312 | conserved hypothetical membrane protein |  | 337 | Unknown |
| TPE_0320 | hypothetical protein |  | 51 | Unknown |
| TPE_0324 (TP0324, TP0325) | treponemal conserved hypothetical outer membrane protein |  | 1468 | Unknown |
| TPE_0333 | conserved hypothetical protein |  | 182 | Unknown |
| TPE_0334 | conserved hypothetical protein |  | 405 | Unknown |
| TPE_0338 | treponemal conserved hypothetical membrane protein |  | 162 | Unknown |
| TPE_0347 | hypothetical membrane protein |  | 276 | Unknown |
| TPE_0348 | treponemal conserved hypothetical membrane protein |  | 374 | Unknown |
| TPE_0352 | conserved hypothetical protein |  | 85 | Unknown |
| TPE_0355 | hypothetical protein |  | 127 | Unknown |
| TPE_0356 | probable RNA-binding protein |  | 89 | Unknown |
| TPE_0358 | conserved hypothetical protein |  | 523 | Unknown |
| TPE_0359 | treponemal conserved hypothetical protein |  | 211 | Unknown |
| TPE_0368 | treponemal conserved hypothetical protein |  | 112 | Unknown |
| TPE_0381 | conserved hypothetical inner membrane protein |  | 238 | Unknown |
| TPE_0385 | treponemal conserved hypothetical protein |  | 124 | Unknown |
| TPE_0392 | conserved hypothetical protein |  | 268 | Unknown |
| TPE_0404 | treponemal conserved hypothetical protein |  | 177 | Unknown |
| TPE_0405 | conserved hypothetical membrane protein |  | 174 | Unknown |
| TPE_0409 | hypothetical protein |  | 61 | Unknown |
| TPE_0412 | conserved hypothetical protein |  | 101 | Unknown |
| TPE_0415 | hypothetical protein |  | 172 | Unknown |
| TPE_0421 | conserved hypothetical protein |  | 683 | Unknown |
| TPE_0423 | conserved hypothetical protein |  | 268 | Unknown |
| TPE_0425 | treponemal conserved hypothetical protein |  | 181 | Unknown |
| TPE_0437 | treponemal conserved hypothetical protein |  | 176 | Unknown |
| TPE_0443 | conserved hypothetical protein |  | 284 | Unknown |
| TPE_0444 | conserved hypothetical protein |  | 342 | Unknown |
| TPE_0449 | treponemal conserved hypothetical protein |  | 196 | Unknown |
| TPE_0454 | treponemal conserved hypothetical protein |  | 229 | Unknown |
| TPE_0455 | conserved hypothetical protein |  | 324 | Unknown |
| TPE_0456 | treponemal conserved hypothetical protein |  | 438 | Unknown |
| TPE_0460 | conserved hypothetical protein |  | 235 | Unknown |
| TPE_0465 | treponemal conserved hypothetical protein |  | 290 | Unknown |
| TPE_0466 | treponemal conserved hypothetical protein |  | 389 | Unknown |
| TPE_0467 | hypothetical protein |  | 82 | Unknown |
| TPE_0468 (TP0468, TP0469) | treponemal conserved hypothetical protein |  | 650 | Unknown |
| TPE_0473 | hypothetical membrane protein |  | 214 | Unknown |
| TPE_0474 | conserved hypothetical protein |  | 245 | Unknown |
| TPE_0479 | hypothetical membrane protein |  | 224 | Unknown |
| TPE_0480 | treponemal conserved hypothetical membrane protein |  | 162 | Unknown |
| TPE_0489 | conserved hypothetical protein |  | 322 | Unknown |
| TPE_0490 | treponemal conserved hypothetical protein |  | 62 | Unknown |
| TPE_0491 | conserved hypothetical protein |  | 351 | Unknown |
| TPE_0494 | conserved hypothetical protein |  | 273 | Unknown |
| TPE_0502 | treponemal conserved hypothetical protein |  | 298 | Unknown |
| TPE_0503 | treponemal conserved hypothetical protein |  | 187 | Unknown |
| TPE_0522 | conserved hypothetical membrane protein |  | 159 | Unknown |
| TPE_0530 | hypothetical protein |  | 208 | Unknown |
| TPE_0534 | hypothetical protein |  | 341 | Unknown |
| TPE_0535 | hypothetical protein |  | 107 | Unknown |
| TPE_0536 | hypothetical protein |  | 138 | Unknown |
| TPE_0552 | treponemal conserved hypothetical protein |  | 177 | Unknown |
| TPE_0553 | conserved hypothetical membrane protein |  | 403 | Unknown |
| TPE_0557 | conserved hypothetical protein |  | 237 | Unknown |
| TPE_0561 | conserved hypothetical protein |  | 306 | Unknown |
| TPE_0563 | treponemal conserved hypothetical protein |  | 137 | Unknown |
| TPE_0567 | conserved hypothetical protein |  | 199 | Unknown |
| TPE_0570 | conserved hypothetical membrane protein |  | 281 | Unknown |
| TPE_0584 | treponemal conserved hypothetical protein |  | 469 | Unknown |
| TPE_0592 | conserved hypothetical protein |  | 500 | Unknown |
| TPE_0597 (TP0597, TP0598) | treponemal conserved hypothetical protein |  | 719 | Unknown |
| TPE_0599 | treponemal conserved hypothetical protein |  | 217 | Unknown |
| TPE_0608 | treponemal conserved hypothetical protein |  | 242 | Unknown |
| TPE_0617 | treponemal conserved hypothetical protein |  | 92 | Unknown |
| TPE_0622 | treponemal conserved hypothetical membrane protein |  | 593 | Unknown |
| TPE_0625 | treponemal conserved hypothetical protein |  | 250 | Unknown |
| TPE_0648 | conserved hypothetical protein |  | 682 | Unknown |
| TPE_0650 | conserved hypothetical protein |  | 160 | Unknown |
| TPE_0651 | conserved hypothetical membrane protein |  | 855 | Unknown |
| TPE_0661 | treponemal conserved hypothetical protein |  | 170 | Unknown |
| TPE_0674 | conserved hypothetical protein |  | 211 | Unknown |
| TPE_0675 | conserved hypothetical protein |  | 332 | Unknown |
| TPE_0676 | hypothetical protein |  | 92 | Unknown |
| TPE_0677 | treponemal conserved hypothetical protein |  | 202 | Unknown |
| TPE_0678 | treponemal conserved hypothetical protein |  | 307 | Unknown |
| TPE_0679 | treponemal conserved hypothetical membrane protein |  | 105 | Unknown |
| TPE_0690 | treponemal conserved hypothetical protein |  | 193 | Unknown |
| TPE_0693 | hypothetical protein |  | 440 | Unknown |
| TPE_0700 | treponemal conserved hypothetical protein |  | 130 | Unknown |
| TPE_0707 | hypothetical membrane protein |  | 159 | Unknown |
| TPE_0708 | hypothetical protein |  | 171 | Unknown |
| TPE_0710 | conserved hypothetical protein |  | 656 | Unknown |
| TPE_0711 | conserved hypothetical protein |  | 190 | Unknown |
| TPE_0739 | conserved hypothetical protein |  | 398 | Unknown |
| TPE_0740 | conserved hypothetical protein |  | 249 | Unknown |
| TPE_0744 | conserved hypothetical protein |  | 108 | Unknown |
| TPE_0747 | treponemal conserved hypothetical protein |  | 344 | Unknown |
| TPE_0749 | hypothetical protein |  | 73 | Unknown |
| TPE_0750 | treponemal conserved hypothetical protein |  | 223 | Unknown |
| TPE_0753 | treponemal conserved hypothetical protein |  | 94 | Unknown |
| TPE_0761 | conserved hypothetical membrane protein |  | 292 | Unknown |
| TPE_0763 | treponemal conserved hypothetical membrane protein |  | 277 | Unknown |
| TPE_0772 | treponemal conserved hypothetical protein |  | 274 | Unknown |
| TPE_0777 | treponemal conserved hypothetical protein |  | 81 | Unknown |
| TPE_0779 | membrane-associated protein DedA |  | 214 | Unknown |
| TPE_0783 | treponemal conserved hypothetical protein |  | 377 | Unknown |
| TPE_0784 | treponemal conserved hypothetical protein |  | 199 | Unknown |
| TPE_0785 | conserved hypothetical protein |  | 228 | Unknown |
| TPE_0788 | treponemal conserved hypothetical protein |  | 296 | Unknown |
| TPE_0791 | hypothetical protein |  | 89 | Unknown |
| TPE_0793 | treponemal conserved hypothetical protein |  | 539 | Unknown |
| TPE_0795 | hypothetical protein |  | 52 | Unknown |
| TPE_0802 | hypothetical protein |  | 134 | Unknown |
| TPE_0803 | treponemal conserved hypothetical protein |  | 402 | Unknown |
| TPE_0816 | treponemal conserved hypothetical protein |  | 221 | Unknown |
| TPE_0818 | hypothetical protein |  | 50 | Unknown |
| TPE_0820 | conserved hypothetical protein |  | 256 | Unknown |
| TPE_0825 | hypothetical protein |  | 72 | Unknown |
| TPE_0826 | conserved hypothetical membrane protein |  | 273 | Unknown |
| TPE_0827 | conserved hypothetical protein |  | 320 | Unknown |
| TPE_0832 | treponemal conserved hypothetical protein |  | 271 | Unknown |
| TPE_0835 | conserved hypothetical protein |  | 934 | Unknown |
| TPE_0839 | hypothetical protein |  | 335 | Unknown |
| TPE_0843 | treponemal conserved hypothetical protein |  | 274 | Unknown |
| TPE_0845 | conserved hypothetical protein |  | 177 | Unknown |
| TPE_0846 | conserved hypothetical protein |  | 108 | Unknown |
| TPE_0847 | treponemal conserved hypothetical protein |  | 111 | Unknown |
| TPE_0867 | hypothetical protein |  | 61 | Unknown |
| TPE_0869 | hypothetical protein |  | 78 | Unknown |
| TPE_0871 | hypothetical protein |  | 53 | Unknown |
| TPE_0873 | treponemal conserved hypothetical protein |  | 204 | Unknown |
| TPE_0874 | conserved hypothetical protein |  | 91 | Unknown |
| TPE_0875 | conserved hypothetical protein |  | 135 | Unknown |
| TPE_0876 | conserved hypothetical protein |  | 221 | Unknown |
| TPE_0877 | conserved hypothetical protein |  | 365 | Unknown |
| TPE_0879 | conserved hypothetical membrane protein |  | 493 | Unknown |
| TPE_0882 | conserved hypothetical protein |  | 500 | Unknown |
| TPE_0883 | conserved hypothetical membrane protein |  | 372 | Unknown |
| TPE_0884 | treponemal conserved hypothetical membrane protein |  | 365 | Unknown |
| TPE_0894 | conserved hypothetical protein |  | 340 | Unknown |
| TPE_0895 | conserved hypothetical protein |  | 168 | Unknown |
| TPE_0896 | hypothetical protein |  | 50 | Unknown |
| TPE_0904 | hypothetical protein |  | 83 | Unknown |
| TPE_0910 | treponemal conserved hypothetical protein |  | 306 | Unknown |
| TPE_0911 | conserved hypothetical protein |  | 83 | Unknown |
| TPE_0914 | conserved hypothetical protein |  | 128 | Unknown |
| TPE_0915 | conserved hypothetical protein |  | 444 | Unknown |
| TPE_0918 | conserved hypothetical membrane protein |  | 257 | Unknown |
| TPE_0923 | hypothetical outer membrane protein |  | 340 | Unknown |
| TPE_0927 | hypothetical protein |  | 95 | Unknown |
| TPE_0930 | treponemal conserved hypothetical protein |  | 573 | Unknown |
| TPE_0931 | conserved hypothetical protein |  | 476 | Unknown |
| TPE_0935 | conserved hypothetical protein |  | 318 | Unknown |
| TPE_0941 | treponemal conserved hypothetical protein |  | 172 | Unknown |
| TPE_0942 | treponemal conserved hypothetical protein |  | 154 | Unknown |
| TPE_0944 | conserved hypothetical protein |  | 390 | Unknown |
| TPE_0948 | Jag family protein |  | 231 | Unknown |
| TPE_0949a | conserved hypothetical protein |  | 94 | Unknown |
| TPE_0956 | conserved hypothetical protein |  | 341 | Unknown |
| TPE_0966 | treponemal conserved hypothetical protein |  | 544 | Unknown |
| TPE_0974 | treponemal conserved hypothetical protein |  | 93 | Unknown |
| TPE_0976 | hypothetical membrane protein |  | 459 | Unknown |
| TPE_0977 | conserved hypothetical protein |  | 286 | Unknown |
| TPE_0981 | conserved hypothetical protein |  | 387 | Unknown |
| TPE_0983 | treponemal conserved hypothetical protein |  | 238 | Unknown |
| TPE_0989 | conserved hypothetical membrane protein |  | 290 | Unknown |
| TPE_0992 | conserved hypothetical protein |  | 95 | Unknown |
| TPE_1000 | hypothetical protein |  | 223 | Unknown |
| TPE_1002 | conserved hypothetical protein |  | 251 | Unknown |
| TPE_1003 | treponemal conserved hypothetical membrane protein |  | 345 | Unknown |
| TPE_1014 | treponemal conserved hypothetical protein |  | 638 | Unknown |
| TPE_1018 | conserved hypothetical protein |  | 504 | Unknown |
| TPE_1029 | treponemal conserved hypothetical protein |  | 228 | Unknown |
| TPE_1032 | conserved hypothetical protein |  | 144 | Unknown |
