# Supplementary material for: Whole Genome Sequences of Three Treponema pallidum ssp. pertenue Strains: Yaws and Syphilis Treponemes Differ in Less than 0.2% of the Genome Sequence
Source: PLoS Negl Trop Dis. 2012 Jan 24;6(1):e1471. doi: 10.1371/journal.pntd.0001471 (PMC3265458; doi:10.1371/journal.pntd.0001471)
Supplement: Table S4 — T. p. ssp. pertenue genes containing major sequence changes encoding proteins with predicted cell function. T. p. ssp. pertenue genes encoding proteins with predicted cell function containing two to five amino acid changes when compared to T. p. ssp. pallidum genes. (DOC) [file pntd.0001471.s004.doc]

**Table S4. TPE genes containing major sequence changes encoding proteins with predicted cell function**

| **Gene** | **Gene name** | **Gene/protein function** | **Functional gene group** | **Type of change in gene/protein^a^** | **Gene expression rate ^b^** | **Remark** | **Z-test Selection Type (p)^e^** |
| --- | --- | --- | --- | --- | --- | --- | --- |
| 0081 | ***coxM*** | xanthine dehydrogenase | General metabolism | 2 aa S | 0.306 | - |  |
| 0102 |  | UvrD/REP family ATP-dependent DNA helicase | DNA replication, repair, recombination | 2 aa S | 0.534 | - |  |
| 0116 | ***uvrB*** | excision endonuclease subunit UvrB | DNA replication, repair, recombination | 2 aa S | 0.405 | - |  |
| 0140 |  | Trk family potassium (K+) transporter, membrane protein | Transport | 2 aa S | 0.349 | - | Purifying (0.041) |
| 0143 |  | ABC superfamily ATP binding cassette transporter, membrane protein | Transport | 4 aa S | 0.448 | - | Positive (0.013) |
| 0164 | ***troB*** | iron (Fe2+)/zinc (Zn2+)/manganese (Mn2+) ABC superfamily ATP binding cassette transporter, ABC protein | Transport | 2 aa S | 0.923 | - |  |
| 0227 | ***cbiO*** | probable cobalt (Co2+) ABC superfamily ATP binding cassette transporter, ABC protein | Transport | 2 aa S | 1.669 | - |  |
| 0241 | ***rpoB*** | DNA-directed RNA polymerase subunit beta | Transcription | 2 aa S | 0.047 | - |  |
| 0303 | ***mutL*** | DNA mismatch repair protein MutL | DNA replication, repair, recombination | 2 aa S | 0.817 | antigen^c^ |  |
| 0313 | ***tprE*** | Tpr protein E | Potential virulence factor | 4 aa S | 0.7 | MSC in Cuniculi A ortholog^d^– originally detected in [72] |  |
| 0344 | ***trcF*** | transcription-repair coupling factor | Transcription | 3 aa S | 0.273 | - |  |
| 0367 | ***smc*** | chromosome segregation ATPase | Cell processes | 2 aa S | 0.707 | - |  |
| 0379 | ***secA*** | Sec family Type I general secretory pathway protein SecA | Transport | 2 aa S | 0.95 | - |  |
| 0429 | ***ntpI*** | two-sector ATPase, V(0) subunit I | Transport | 2 aa S | 1.166 | - |  |
| 0446 | ***gcpE*** | 4-hydroxy-3-methylbut-2-en-1-yl diphosphate synthase | General metabolism | 2 aa S | 0.338 | - |  |
| 0451a |  | NCS2 family nucleobase:cation symporter-2 | Transport | 2 aa S | - | - |  |
| 0506 | ***tig*** | peptidylprolyl isomerase | Cell processes | 3 aa S | 1.058 | - | Positive (0.036) |
| 0556 | ***asnA*** | aspartate--ammonia ligase | General metabolism | 3 aa S, protein is 4 aa longer (N-end) | 1.813 | - | Positive (0.034) |
| 0559 | ***thiI*** | thiamine biosynthesis ATP pyrophosphatase | General metabolism | 2 aa S | 0.907 | - | Positive (0.020) |
| 0569 | ***pepP*** | Xaa-Pro aminopeptidase | General metabolism | 2 aa S | 0.434 | - |  |
| 0586 | ***leuS*** | leucine--tRNA ligase | Translation | 2 aa S | 0.972 | - |  |
| 0610 | ***tprH*** | Tpr protein H | Potential virulence factor | 2 aa S | 0.916 | - |  |
| 0639 | ***mcp*** | methyl-accepting chemotaxis protein | Cell processes | 3 aa S | 0.726 | - |  |
| 0691 | ***scpA*** | segregation and condensation protein ScpA | Cell processes | 3 aa S | 0.289 | - |  |
| 0695 | ***accC*** | biotin carboxylase | General metabolism | 2 aa S | 0.699 | - |  |
| 0715 | ***flhB*** | IIISP family Type III (virulence-related) secretory pathway protein | Virulence | 2 aa S | 0.171 | - |  |
| 0729 | ***fliK*** | flagellar hook-length control protein FliK | Cell structure | 5 aa S | 2.949 | - | Positive (0.010) |
| 0735 | ***gltD*** | glutamate synthase (NADPH) | General metabolism | 2 aa S | 1.338 | MSC in Cuniculi A ortholog |  |
| 0771 | ***nptA*** | PnaS family phosphate:sodium (Na+) symporter | Transport | 3 aa S | 0.514 | - | Positive (0.042) |
| 0780 | ***nadE*** | NAD(+) synthase | General metabolism | 2 aa S | 0.398 | - |  |
| 0831 | ***argS*** | arginine--tRNA ligase | Translation | 3 aa S | 0.459 | - | Positive (0.050) |
| 0861 | ***glmS*** | glutamine--fructose-6-phosphate transaminase (isomerizing) | General metabolism | 5 aa S | 0.973 | - |  |
| 0898 | ***recB*** | exodeoxyribonuclease V beta subunit | DNA replication, repair, recombination | 5 aa S | 0.413 | MSC in Cuniculi A ortholog |  |
| 0934 |  | DAACS family dicarboxylate/amino acid:sodium (Na+) or proton (H+) symporter | Transport | 2 aa S | 0.879 | - |  |
| 0949 | ***oxaA*** | Oxa1 family cytochrome oxidase biogenesis protein | Transport | 3 aa S | 0.492 | - |  |
| 0988 | ***marC*** | MarC family multiple antibiotic resistance transporter | Transport | 2 aa S | 0.653 | - |  |

*T. p.* ssp. *pertenue* (TPE) genes encoding proteins with predicted cell function containing six or more amino acid changes and/or major sequence changes between all studied *T. p.* ssp. *pertenue* and all *T. p.* ssp. *pallidum* strains are shown.

^a^S, substitution; D, deletion; I, insertion

^b^Gene expression rate in Nichols strain grown in rabbits. The gene expression rates were taken from[58].

^c^The corresponding protein was identified as an antigen [54].

^d^The gene was shown to contain frameshift mutations or MSC in the genome of *Treponema paraluiscuniculi* Cuniculi A [33].

^e^The selection test was calculated using the Kumar model [47] using MEGA4 [48] software.
