# Supplementary material for: Whole Genome Sequences of Three Treponema pallidum ssp. pertenue Strains: Yaws and Syphilis Treponemes Differ in Less than 0.2% of the Genome Sequence
Source: PLoS Negl Trop Dis. 2012 Jan 24;6(1):e1471. doi: 10.1371/journal.pntd.0001471 (PMC3265458; doi:10.1371/journal.pntd.0001471)
Supplement: Table S5 — T. p. ssp. pertenue genes containing major sequence changes encoding hypothetical proteins. T. p. ssp. pertenue genes encoding proteins with unknown cell function containing two to five amino acid changes when compared to T. p. ssp. pallidum genes. (DOC) [file pntd.0001471.s005.doc]

**Table S5. TPE genes containing major sequence changes encoding hypothetical proteins**

| **Gene** | **Protein prediction** | **Type of gene/protein change^a^** | **Gene expression^b^** | **Remark** | **Z-test Selection Type (p)^e^** |
| --- | --- | --- | --- | --- | --- |
| 0067 | CHP | 2 aa S, 3 aa D | 0.622 | - |  |
| 0086 | CHP | 3 aa S | 2.497 | - | Positive (0.036) |
| 0110 | TCHP | 5 aa S | 0.561 | - |  |
| 0335 | TCHMP | 2 aa S | 0.882 | - |  |
| 0346 | CHP | 5 aa S | 2.874 | - | Positive (0.035) |
| 0369 | TCHP | 3 aa S | 0.918 | antigen^c^ |  |
| 0376 | CHP | 2 aa S | 1.587 | - |  |
| 0408 | CHP | 2 aa S | 1.606 | - |  |
| 0422 | TCHP | 2 aa S | 0.511 | - |  |
| 0484 | CHP | 3 aa S | 0.489 | - |  |
| 0515 | CHOMP | 2 aa S | 0.104 | MSC in Cuniculi A ortholog^d^ | Positive (0.000) |
| 0548 | TCHMP | 2 aa S, 2 aa D | 0.898 | MSC in Cuniculi A ortholog |  |
| 0564 | TCHMP | 2 aa S | 0.378 | - |  |
| 0618 | TCHP | 2 aa S | 1.227 | antigen, MSC in Cuniculi A ortholog |  |
| 0638 | TCHMP | 2 aa S | 0.711 | - |  |
| 0697 | HMP | 5 aa S | 0.885 | - |  |
| 0698 | HMP | 2 aa S | 1.813 | MSC in Cuniculi A ortholog | Positive (0.046) |
| 0738 | CHP | 2 aa S | 0.709 | - |  |
| 0854 | CHMP | 2 aa S | 0.221 | - |  |
| 0856 | TCHP | 3 aa S | 5.697 | antigen, MSC in Cuniculi A ortholog |  |
| 0859 TP0859-60 | TCHP | 2 aa S, 3 aa I, | 2.231 1.204 | MSC in Cuniculi A ortholog |  |
| 0864 | CHP | 2 aa S | 1.100 | - |  |
| 0866 | CHP | 3 aa S | 0.721 | - |  |
| 0967 | TCHP | 3 aa S | 2.844 | MSC in Cuniculi A ortholog |  |
| 0969 | TCHOMP | 5 aa S | 2.751 | MSC in Cuniculi A ortholog |  |
| 0987 | TCHP | 2 aa S | 0.555 | - |  |
| 0993 | possible rare lipoprotein A -CHP | 3 aa S | 2.487 | antigen | Positive (0.033) |

*T. p.* ssp. *pertenue* (TPE) genes encoding hypothetical proteins containing six or more amino acid changes and/or major sequence changes between all studied *T. p.* ssp. *pertenue* and all *T. p.* ssp. *pallidum* strains are shown. CHP, conserved hypothetical protein; CHMP, conserved hypothetical membrane protein; CHOMP, conserved hypothetical outer membrane protein; HP, hypothetical protein; HMP, hypothetical membrane protein; TCHMP, treponemal conserved hypothetical membrane protein; TCHOMP, treponemal conserved hypothetical outer membrane protein; TCHP, treponemal conserved hypothetical protein.

^a^S, substitution; D, deletion; I, insertion

^b^Gene expression rate in Nichols strain grown in rabbits. The gene expression rates were taken from [58].

^c^The corresponding protein was identified as an antigen [54].

^d^The gene was shown to contain frameshift mutations or MSC in the genome of *Treponema paraluiscuniculi* Cuniculi A [33].

^e^The selection test was calculated using the Kumar model [47] using MEGA4 [48] software.
